# Supplementary material for: Notch signaling functions in noncanonical juxtacrine manner in platelets to amplify thrombogenicity
Source: eLife. 2022 Oct 3;11:e79590. doi: 10.7554/eLife.79590 (PMC9629830; doi:10.7554/eLife.79590)
Supplement: Figure 1—source data 2. [file elife-79590-fig1-data2.zip › Figure 1-source data 2 (labeled blot).pptx]

## Slide 1
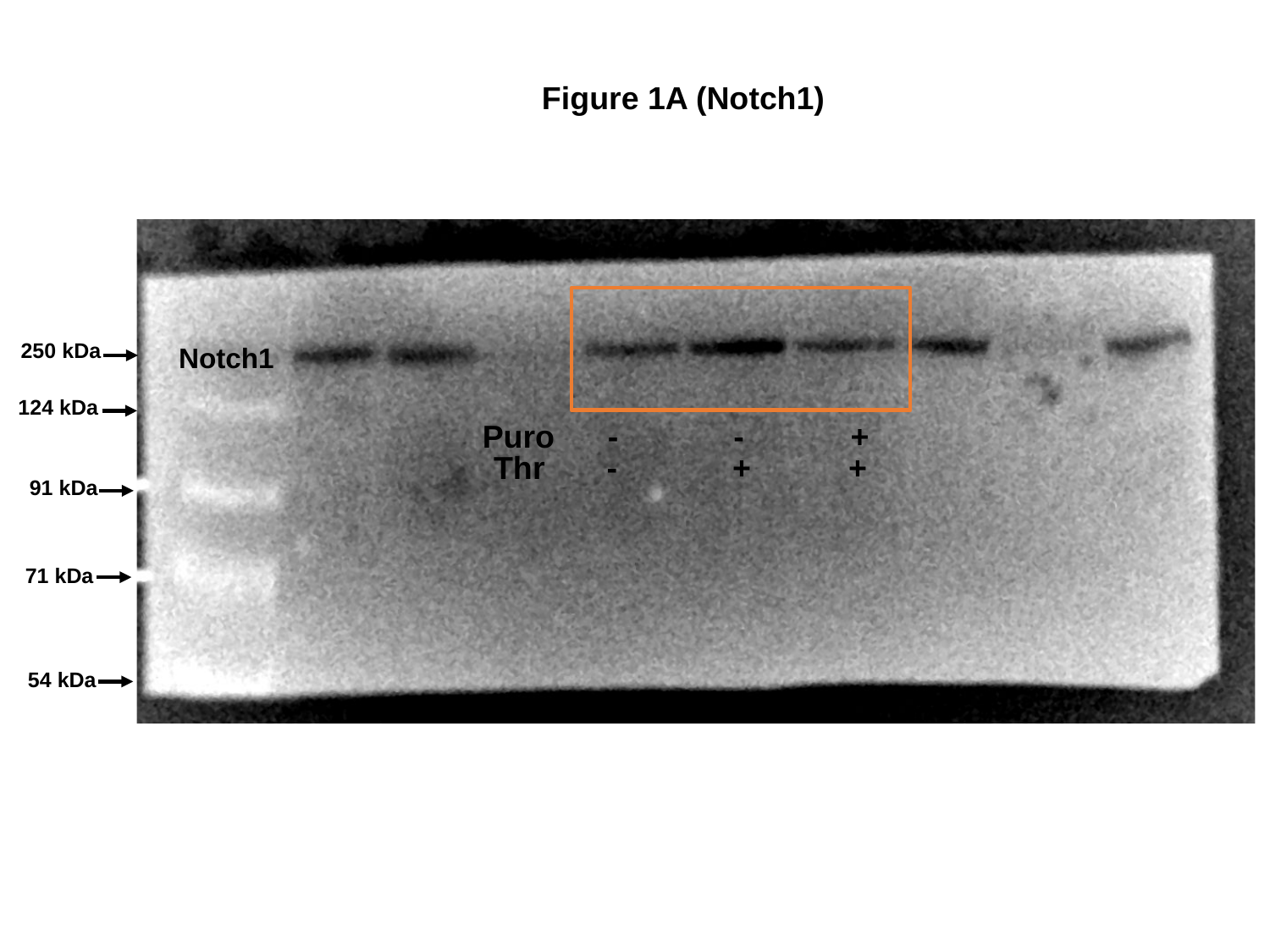

Figure 1A (Notch1)
250 kDa
Notch1
124 kDa
Puro - - +
Thr - + +
91 kDa
71 kDa
54 kDa

## Slide 2
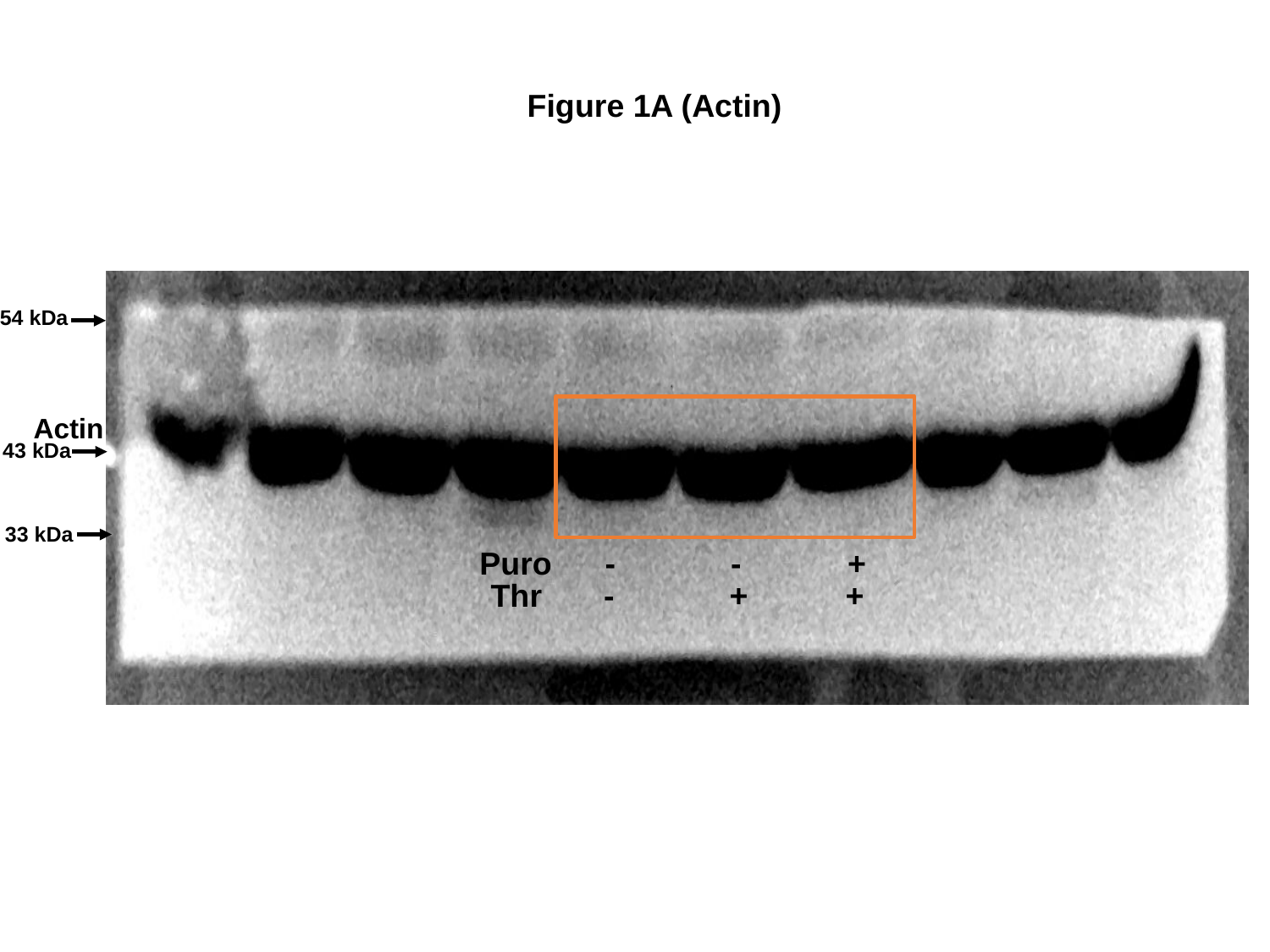

Figure 1A (Actin)
54 kDa
Actin
43 kDa
33 kDa
Puro - - +
Thr - + +

## Slide 3
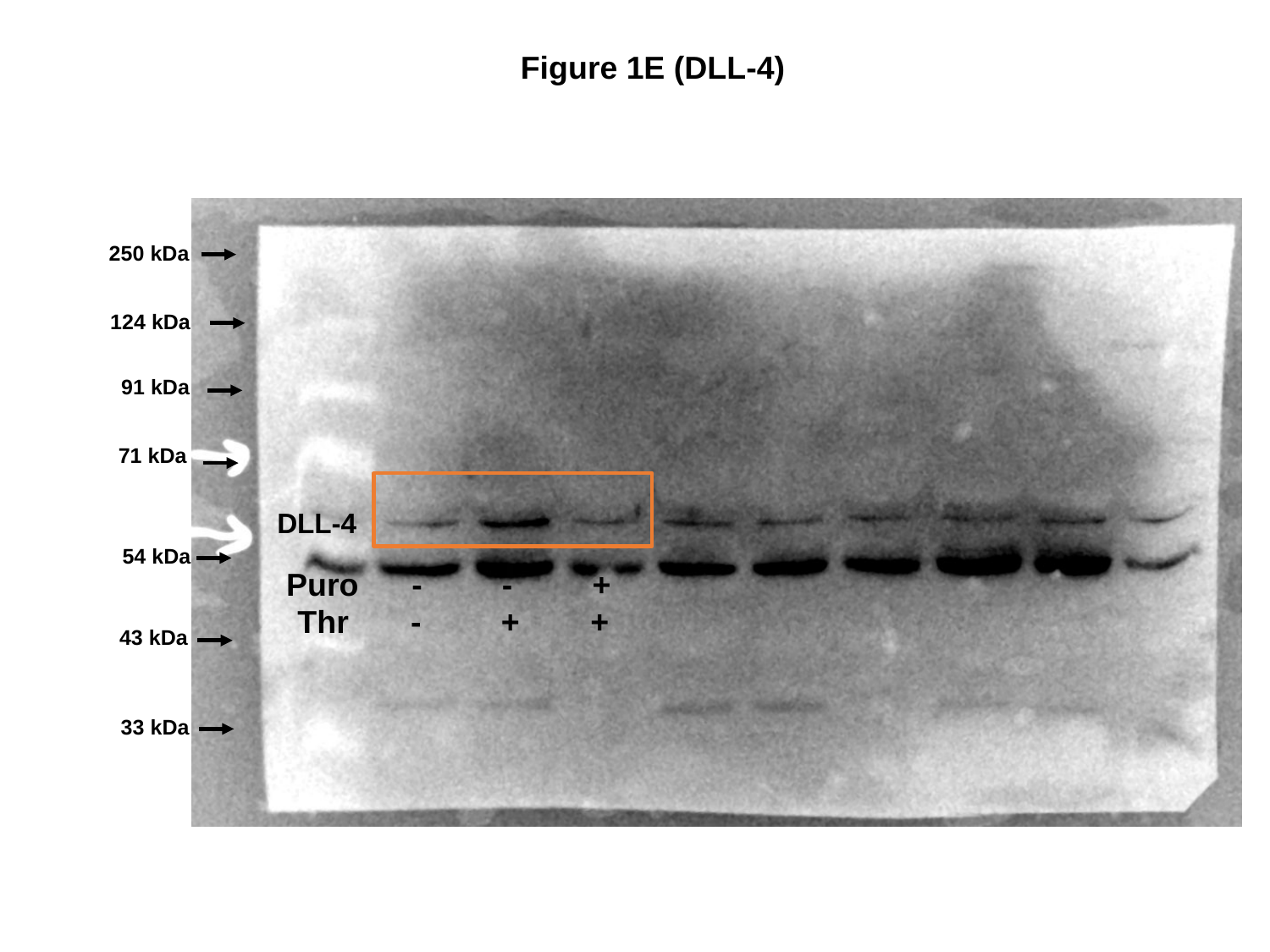

Figure 1E (DLL-4)
250 kDa
124 kDa
91 kDa
71 kDa
DLL-4
54 kDa
Puro - - +
Thr - + +
43 kDa
33 kDa

## Slide 4
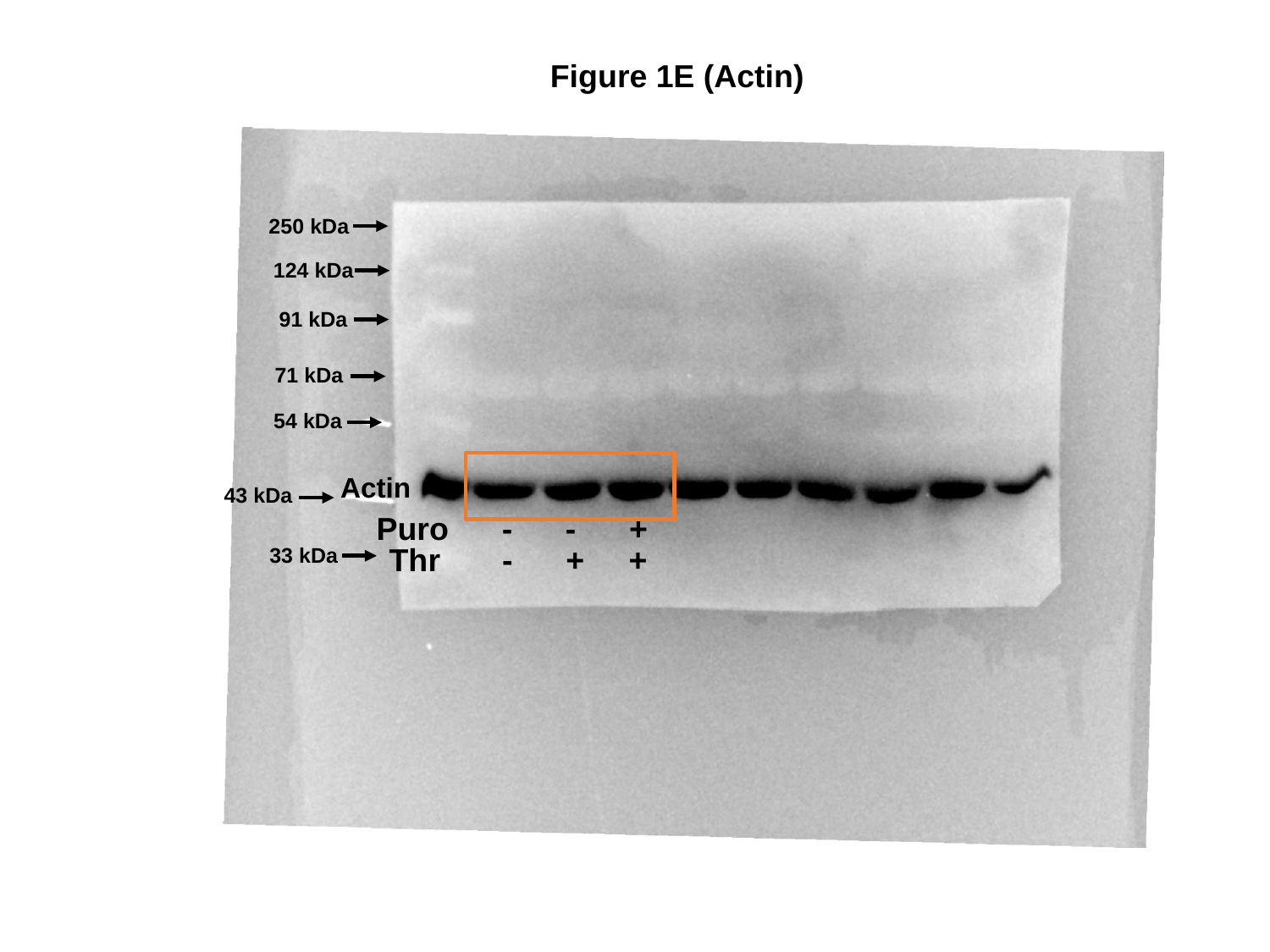

Figure 1E (Actin)
250 kDa
124 kDa
91 kDa
71 kDa
54 kDa
Actin
43 kDa
Puro - - +
Thr - + +
33 kDa
