# Supplementary figures and images for: Notch signaling functions in noncanonical juxtacrine manner in platelets to amplify thrombogenicity
Source: eLife. 2022 Oct 3;11:e79590. doi: 10.7554/eLife.79590 (PMC9629830; doi:10.7554/eLife.79590)

## Slide 1
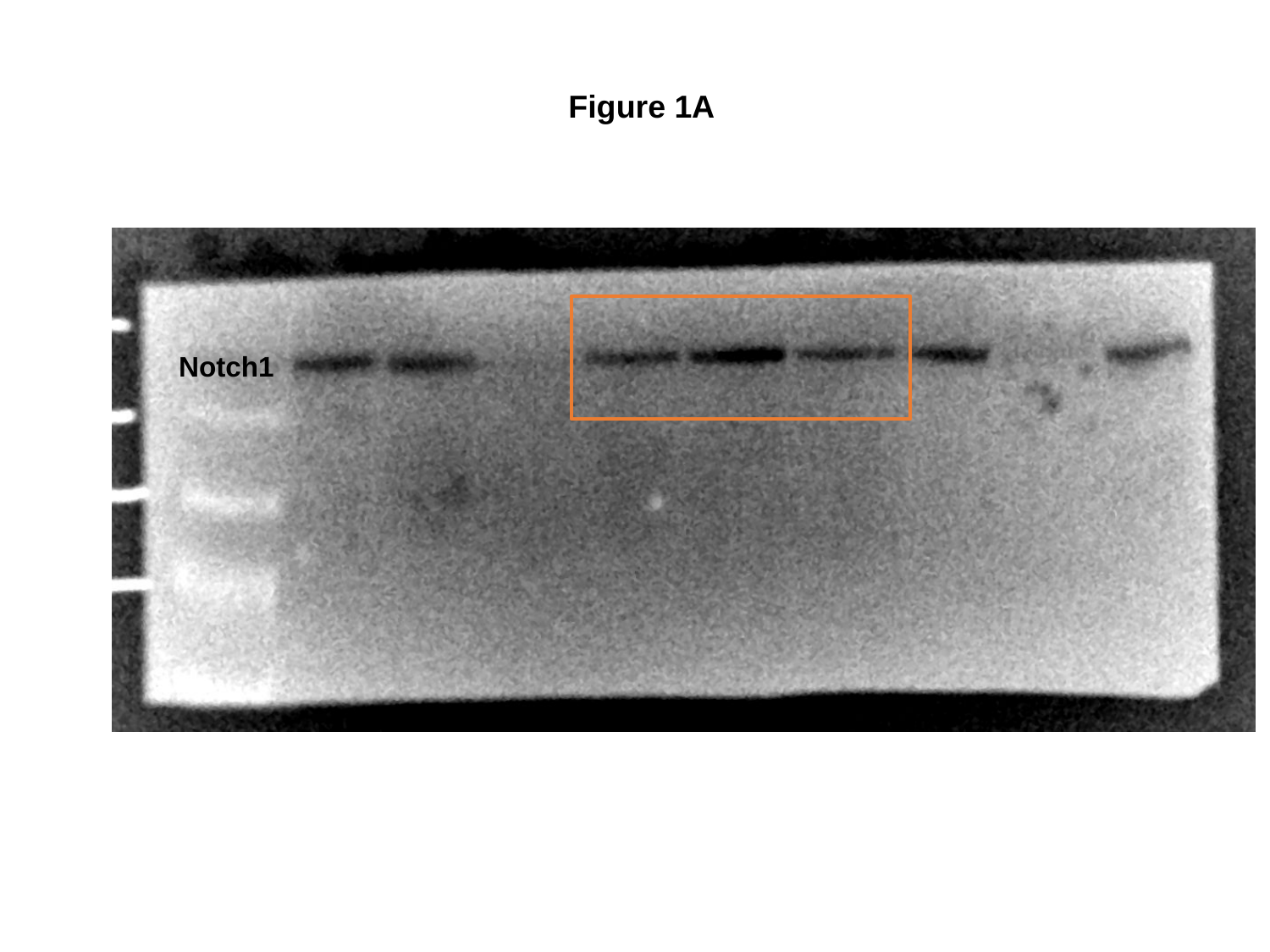

Figure 1A
Notch1

## Slide 2
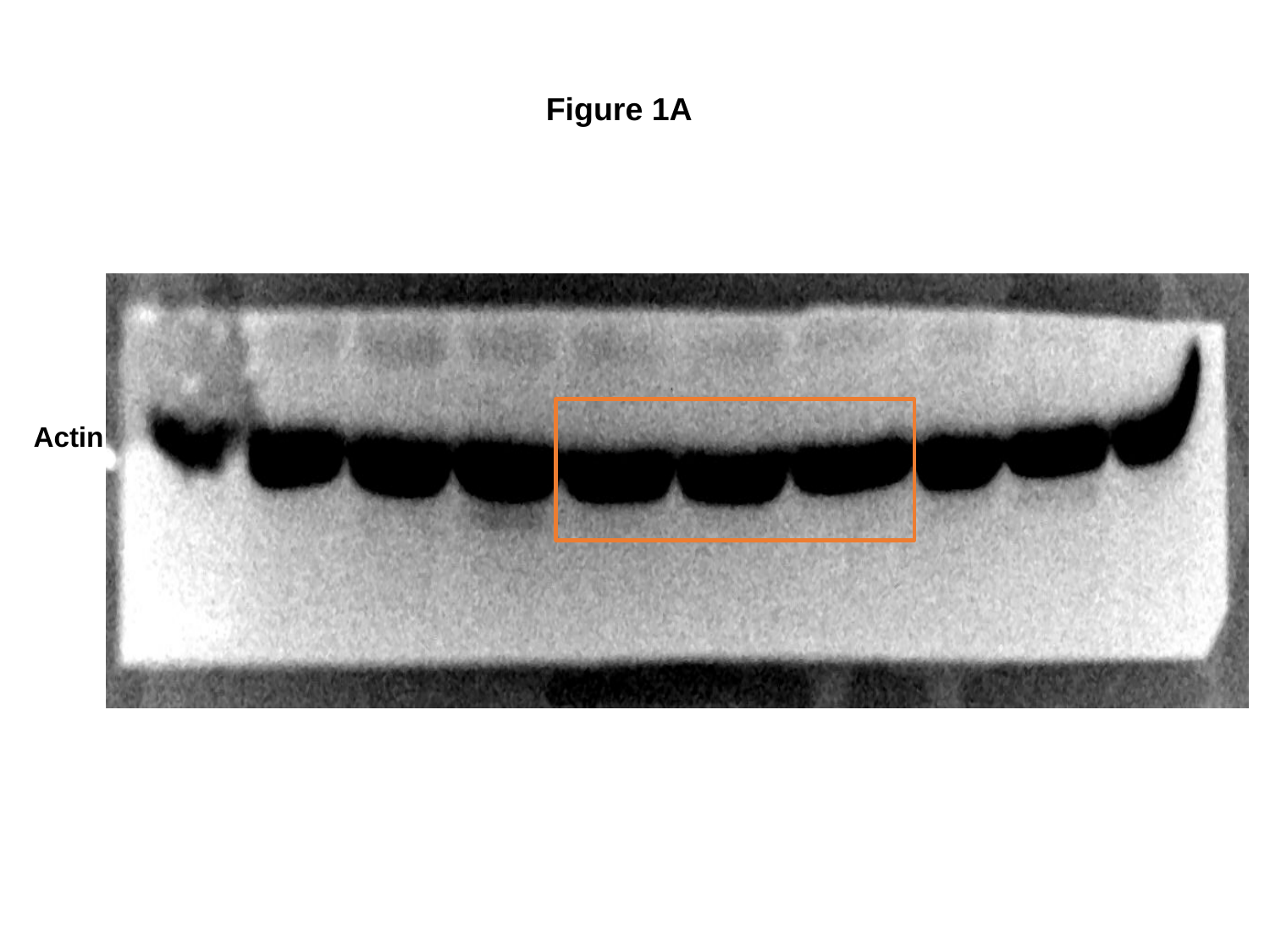

Figure 1A
Actin

## Slide 3
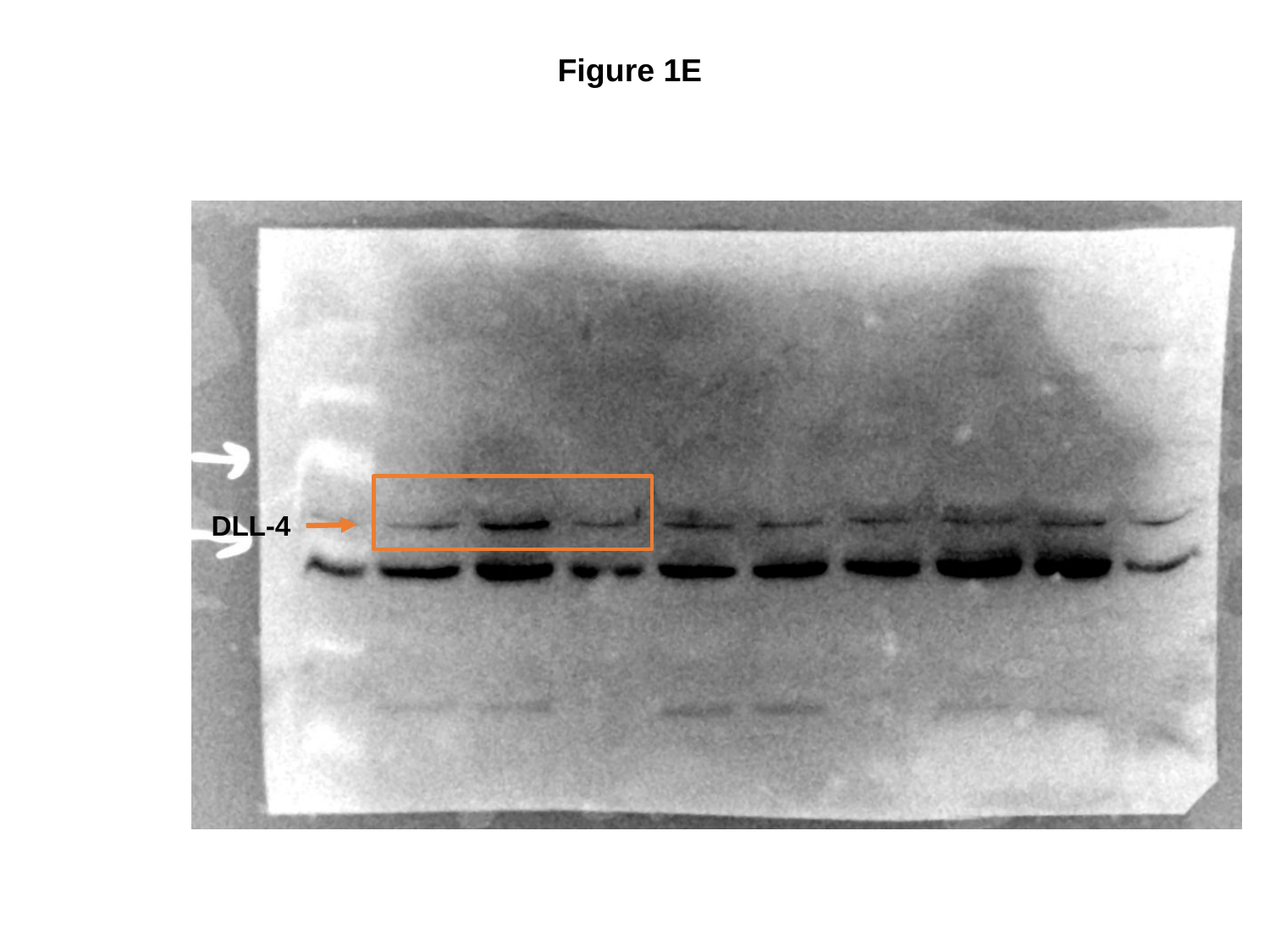

Figure 1E
DLL-4

## Slide 4
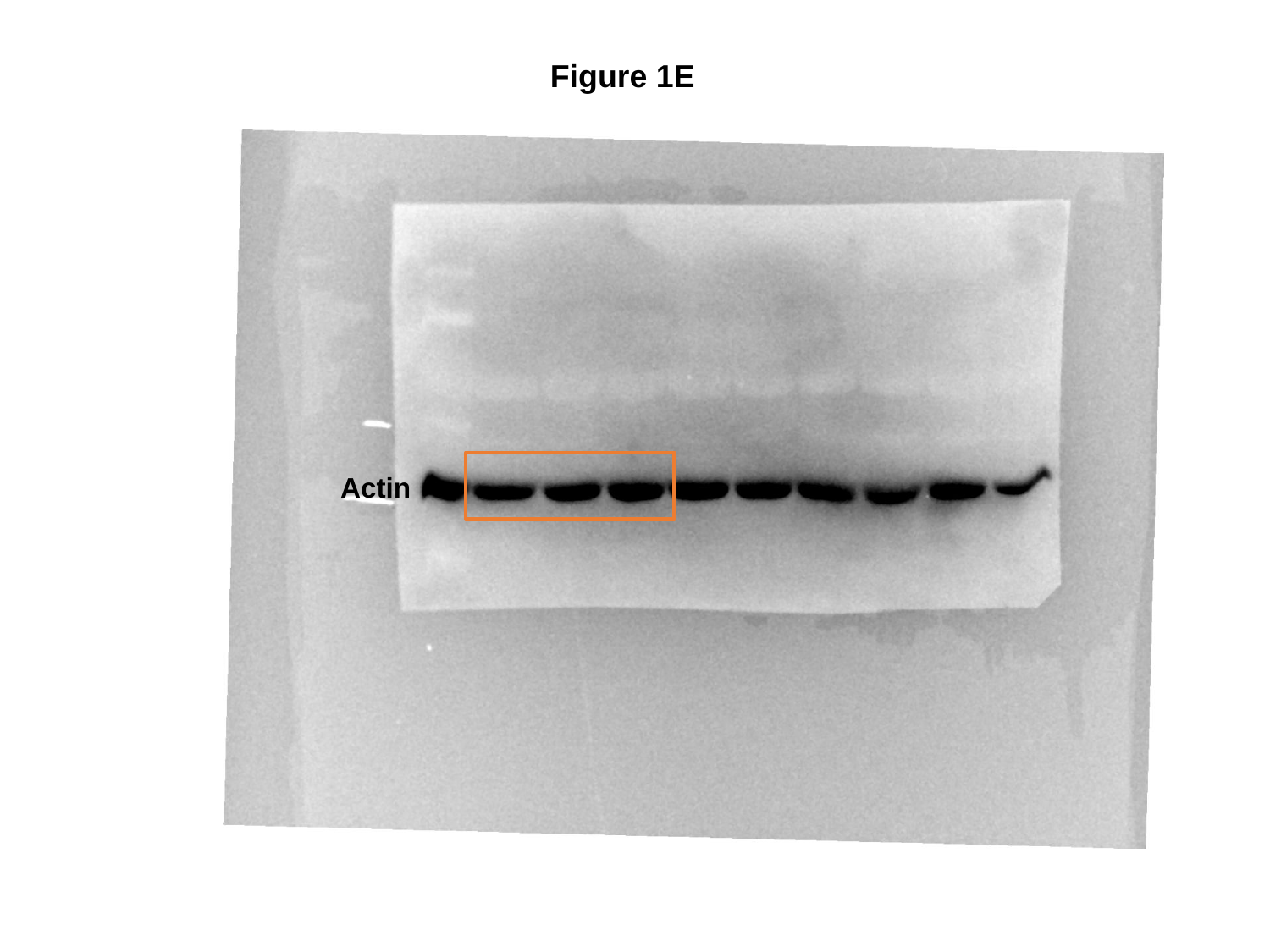

Figure 1E
Actin

Supplement: Figure 1—source data 3. [file elife-79590-fig1-data3.zip › Figure 1-source data 2 (Unedited blot).pptx]
