# Supplementary material for: Notch signaling functions in noncanonical juxtacrine manner in platelets to amplify thrombogenicity
Source: eLife. 2022 Oct 3;11:e79590. doi: 10.7554/eLife.79590 (PMC9629830; doi:10.7554/eLife.79590)
Supplement: Figure 2—source data 2. [file elife-79590-fig2-data2.zip › FIgure 2-source data 2 (Labeled blot).pptx]

## Slide 1
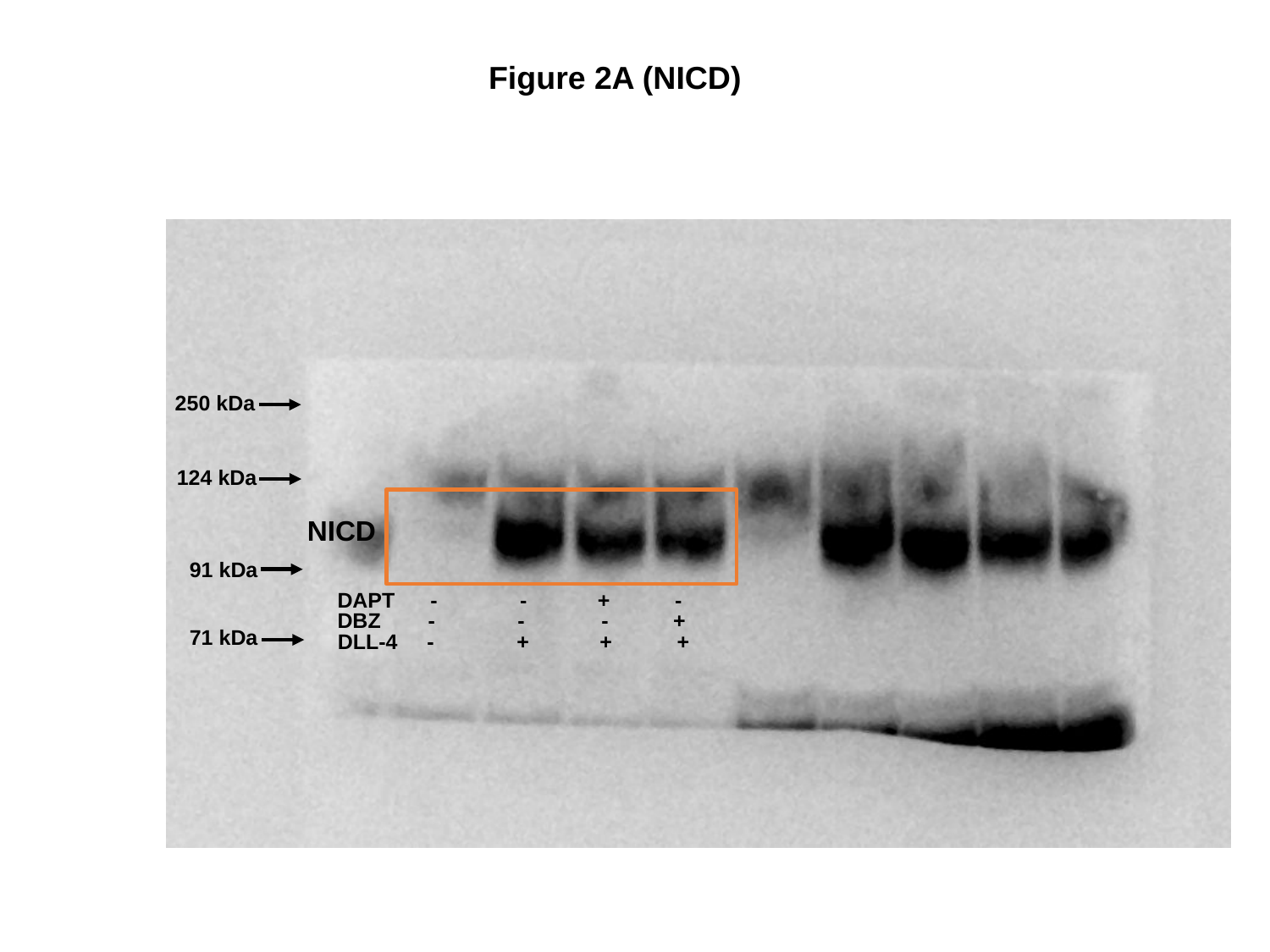

Figure 2A (NICD)
250 kDa
124 kDa
NICD
91 kDa
DAPT - - + -
DBZ - - - +
DLL-4 - + + +
71 kDa

## Slide 2
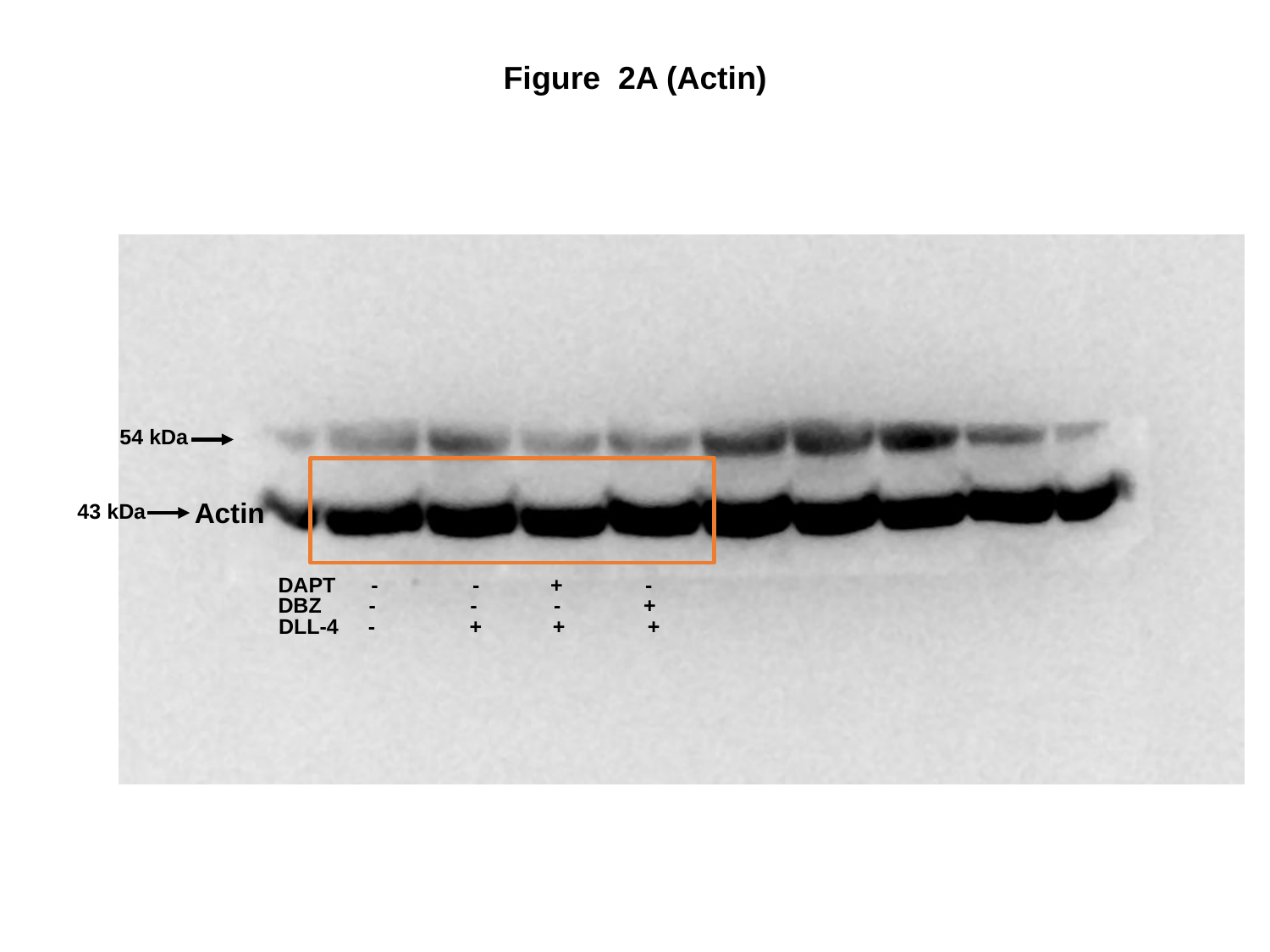

Figure 2A (Actin)
54 kDa
Actin
43 kDa
DAPT - - + -
DBZ - - - +
DLL-4 - + + +

## Slide 3
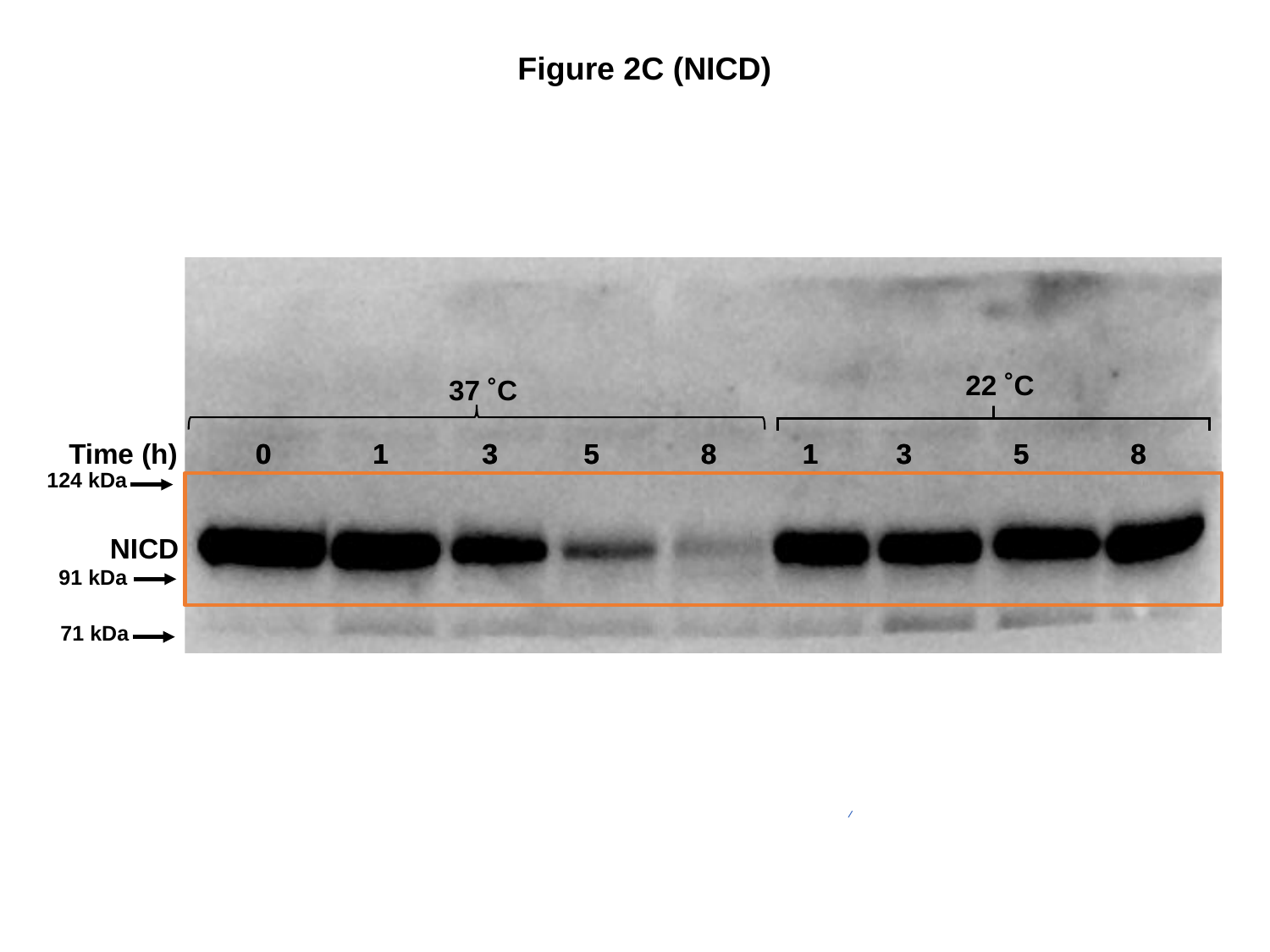

Figure 2C (NICD)
22 ˚C
37 ˚C
Time (h)
 0 1 3 5 8 1 3 5 8
 0 1 3 5 8 1 3 5 8
124 kDa
NICD
91 kDa
71 kDa

## Slide 4
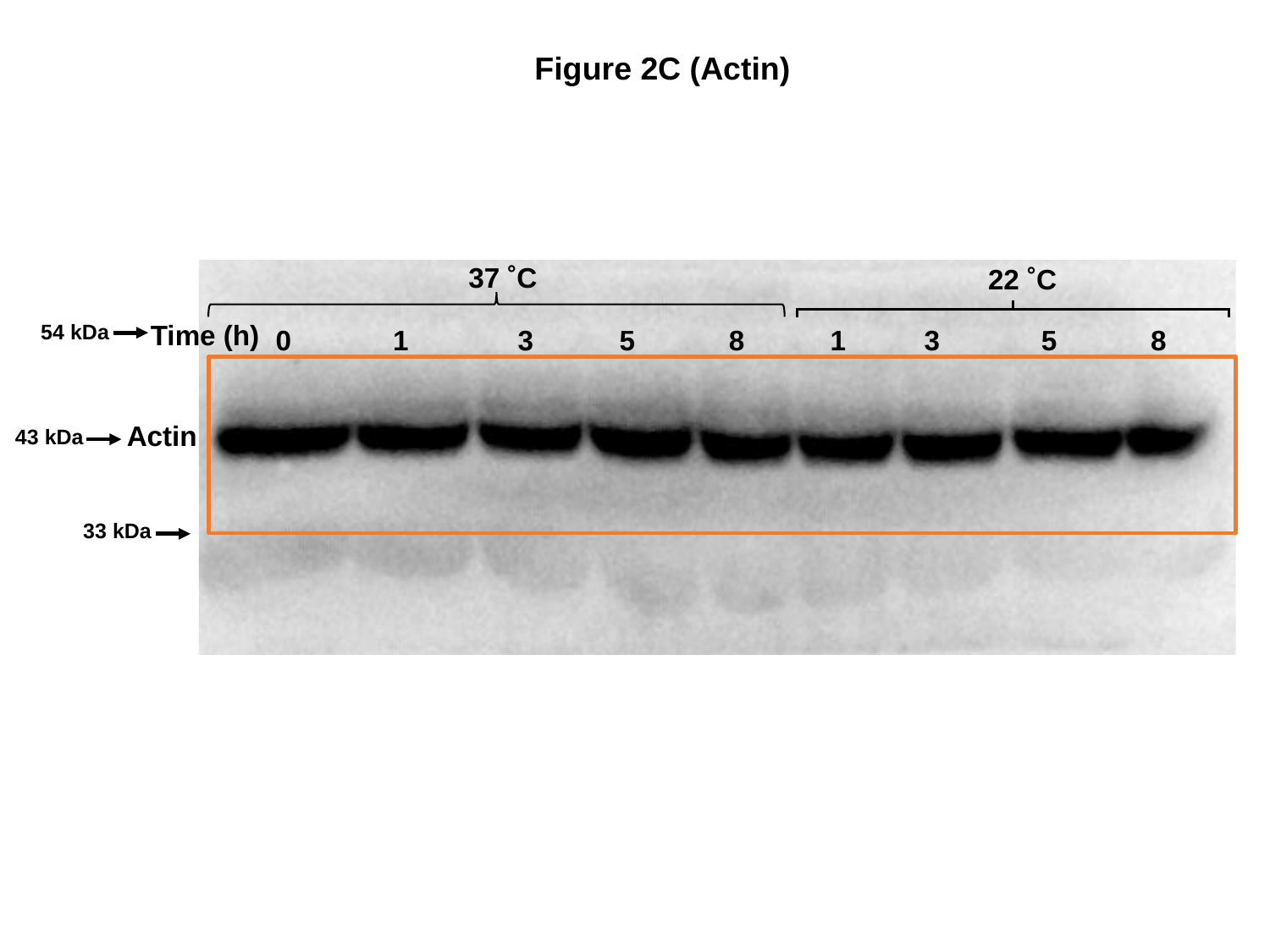

Figure 2C (Actin)
37 ˚C
22 ˚C
Time (h)
 0 1 3 5 8 1 3 5 8
54 kDa
Actin
43 kDa
33 kDa

## Slide 5
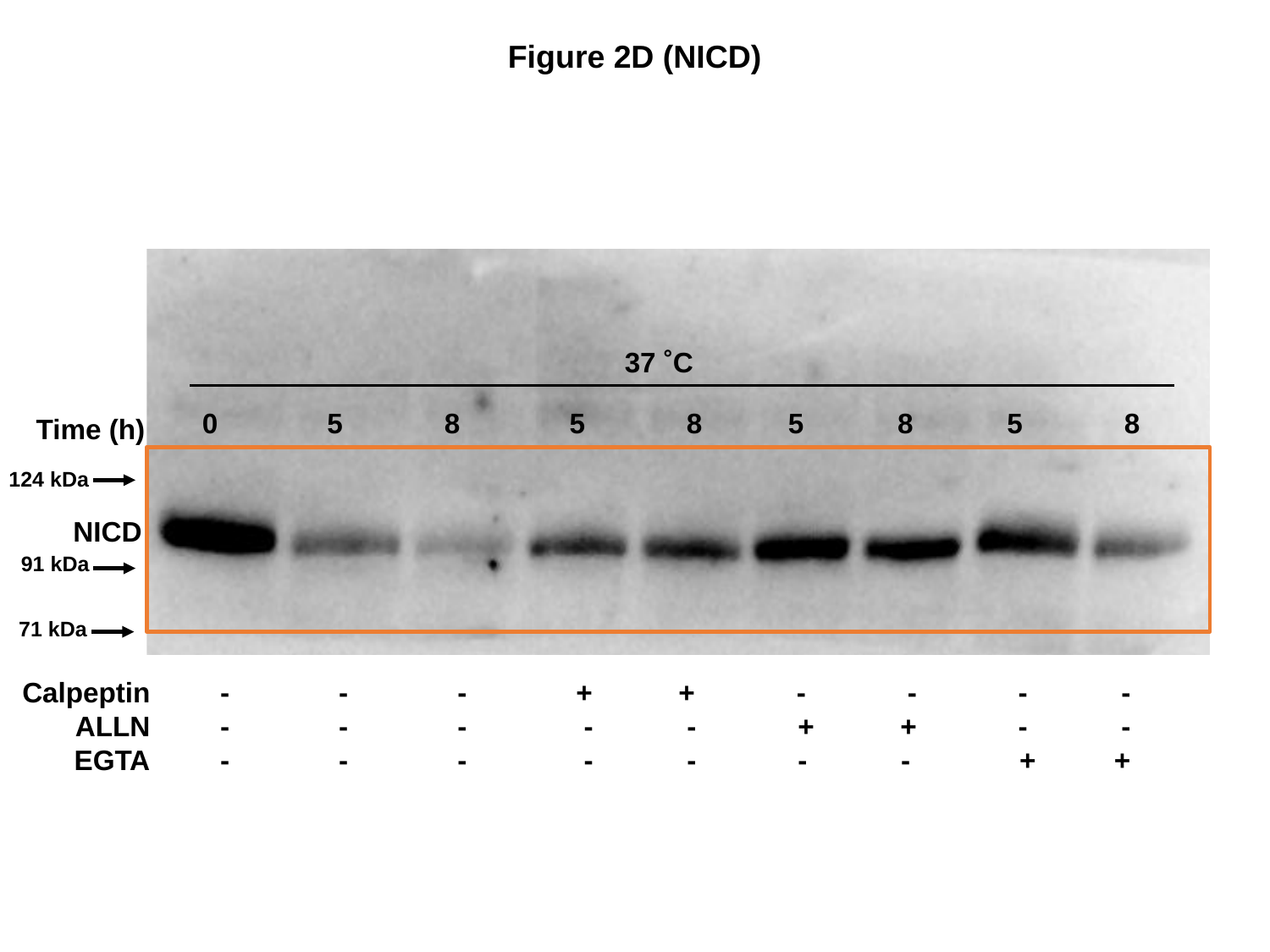

Figure 2D (NICD)
37 ˚C
0 5 8 5 8 5 8 5 8
Time (h)
Calpeptin
ALLN
EGTA
 - - - + + - - - -
 - - - - - + + - -
 - - - - - - - + +
124 kDa
NICD
91 kDa
71 kDa

## Slide 6
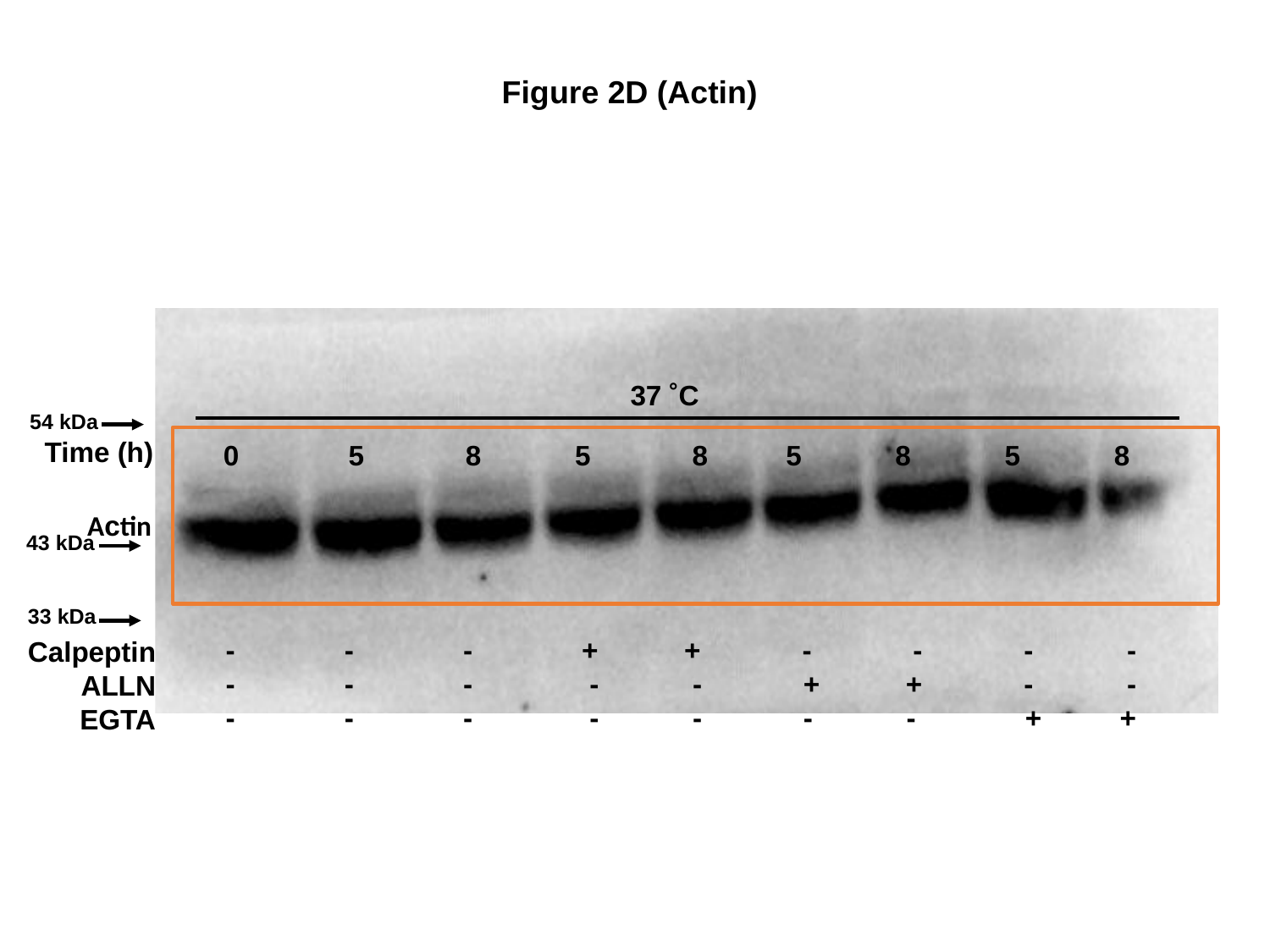

Figure 2D (Actin)
37 ˚C
Time (h)
 0 5 8 5 8 5 8 5 8
 - - - + + - - - -
 - - - - - + + - -
 - - - - - - - + +
Calpeptin
ALLN
EGTA
54 kDa
Actin
43 kDa
33 kDa

## Slide 7
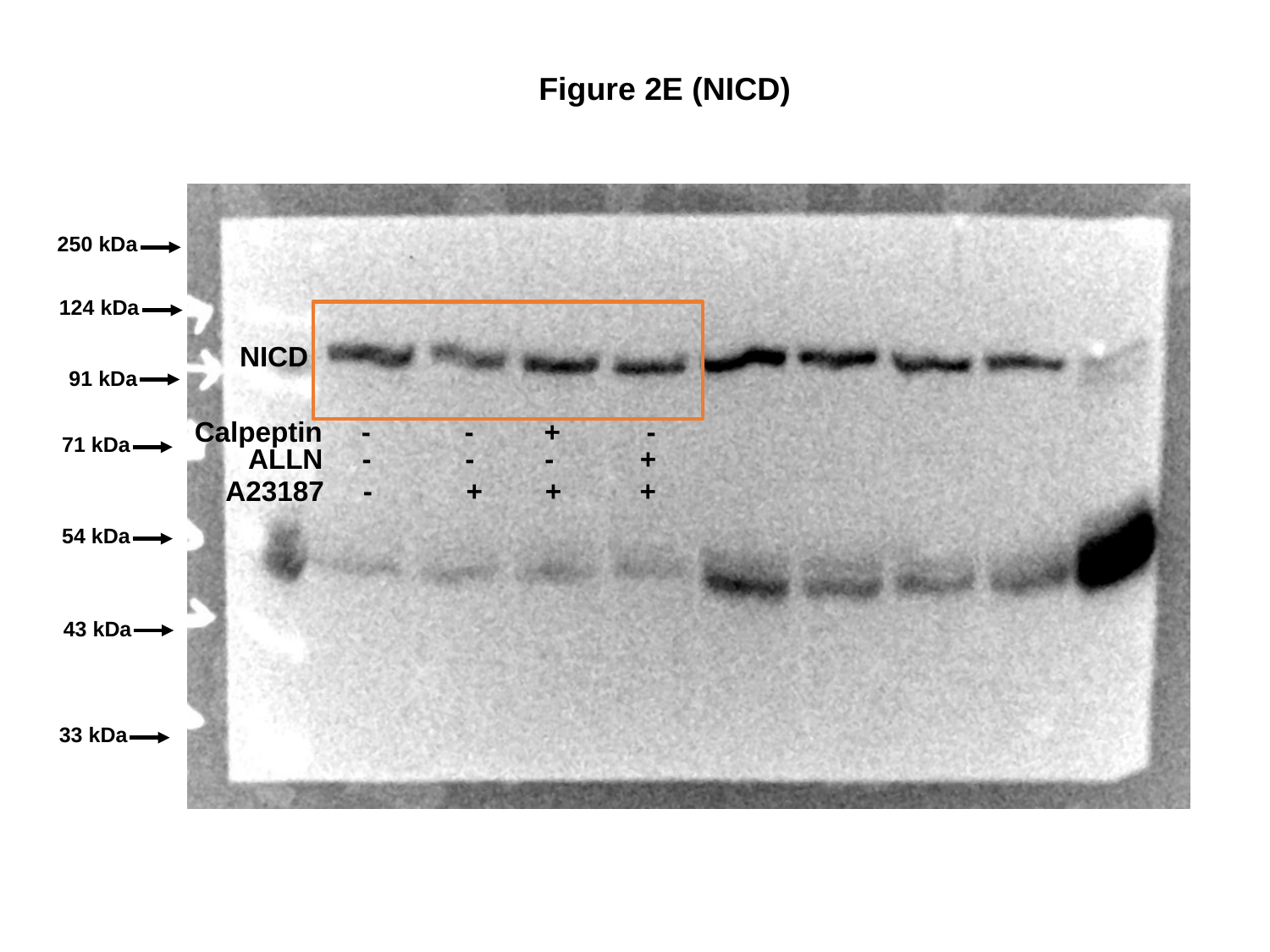

Figure 2E (NICD)
250 kDa
124 kDa
NICD
91 kDa
Calpeptin - - + -
ALLN - - - +
A23187 - + + +
71 kDa
54 kDa
43 kDa
33 kDa

## Slide 8
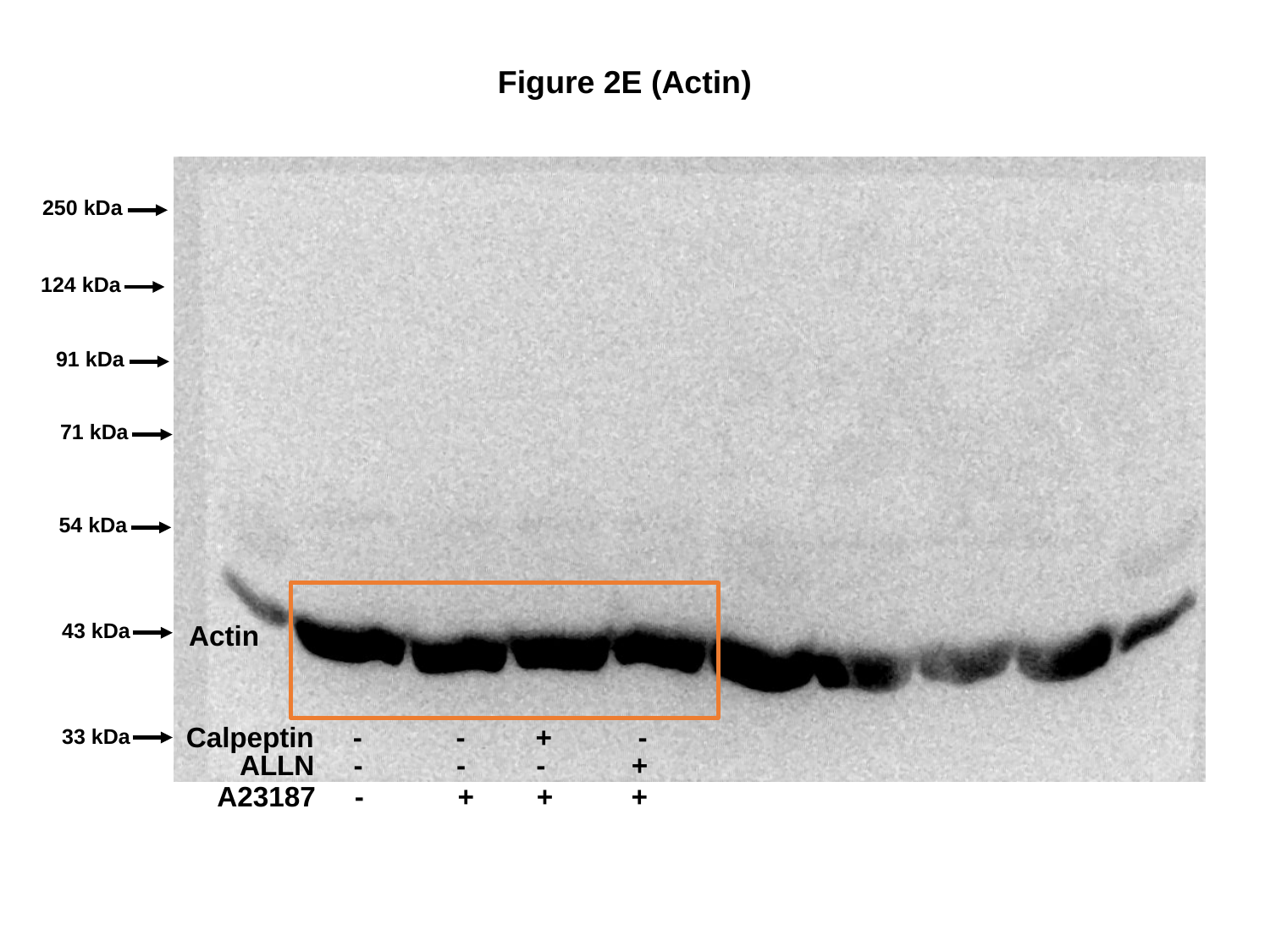

Figure 2E (Actin)
250 kDa
124 kDa
91 kDa
71 kDa
54 kDa
43 kDa
Actin
Calpeptin - - + -
ALLN - - - +
A23187 - + + +
33 kDa
