# Supplementary material for: Notch signaling functions in noncanonical juxtacrine manner in platelets to amplify thrombogenicity
Source: eLife. 2022 Oct 3;11:e79590. doi: 10.7554/eLife.79590 (PMC9629830; doi:10.7554/eLife.79590)
Supplement: Figure 2—source data 3. [file elife-79590-fig2-data3.zip › Figure 2-source data 2 (unedited blot).pptx]

## Slide 1
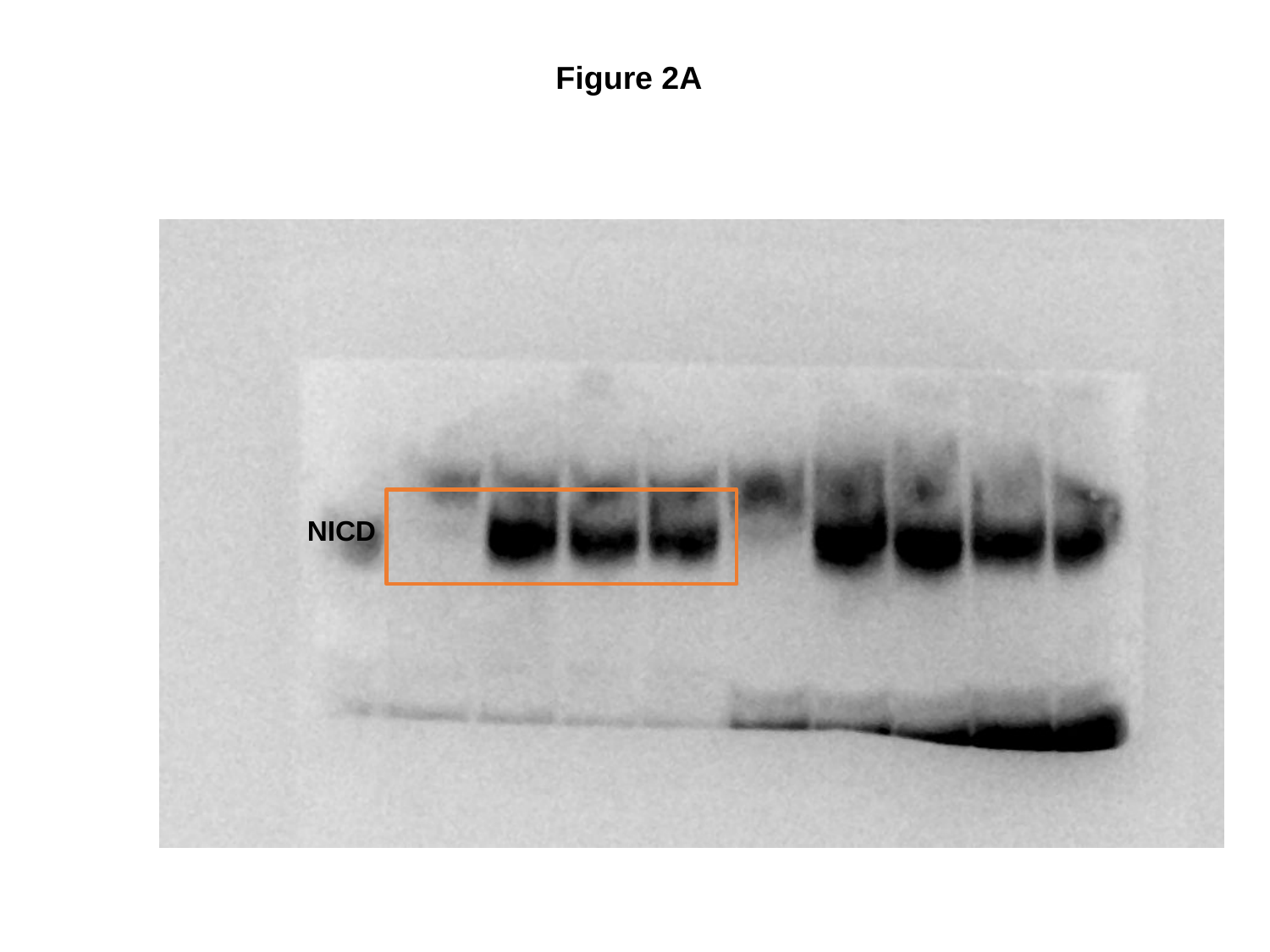

Figure 2A
NICD

## Slide 2
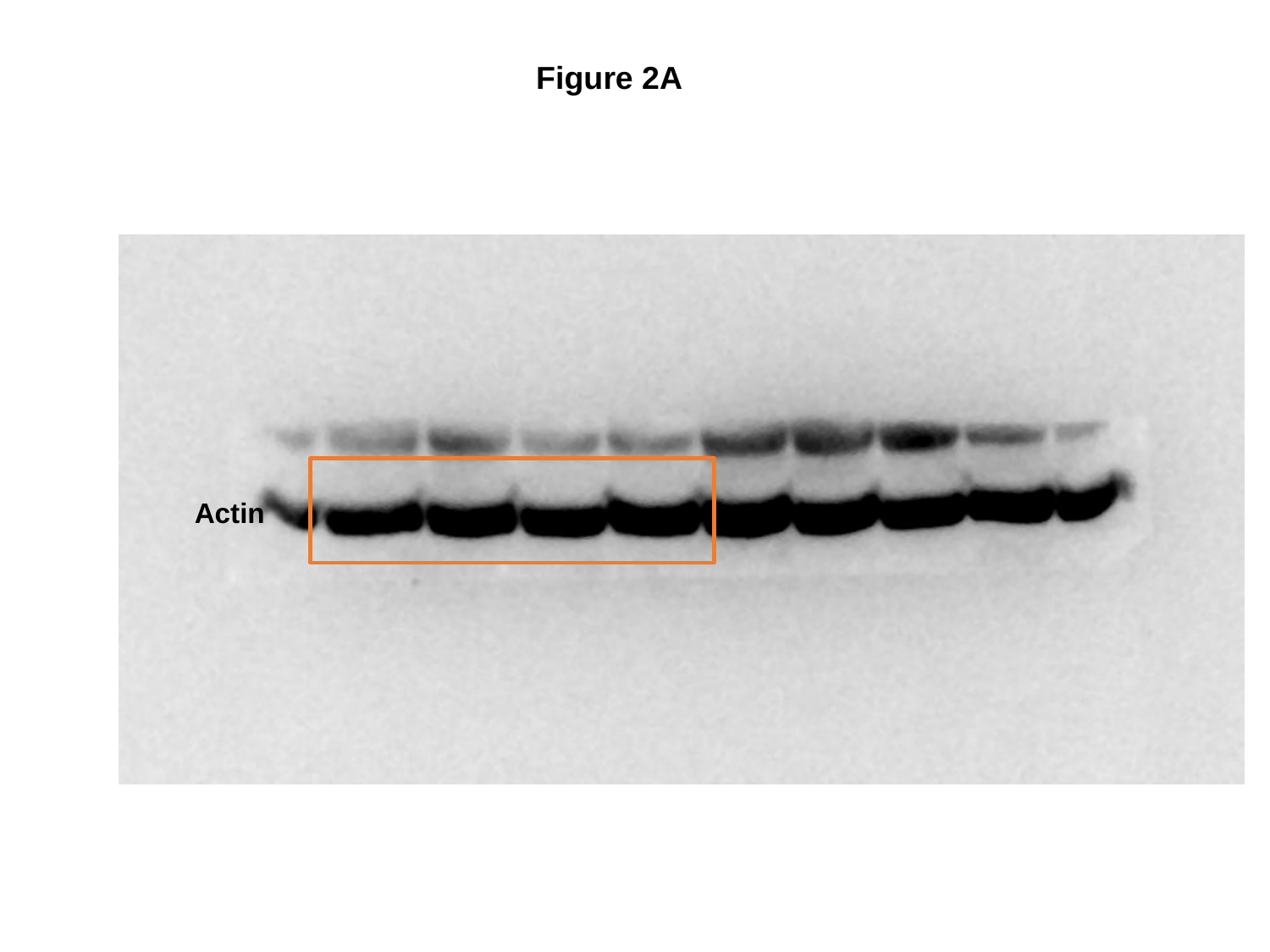

Figure 2A
Actin

## Slide 3
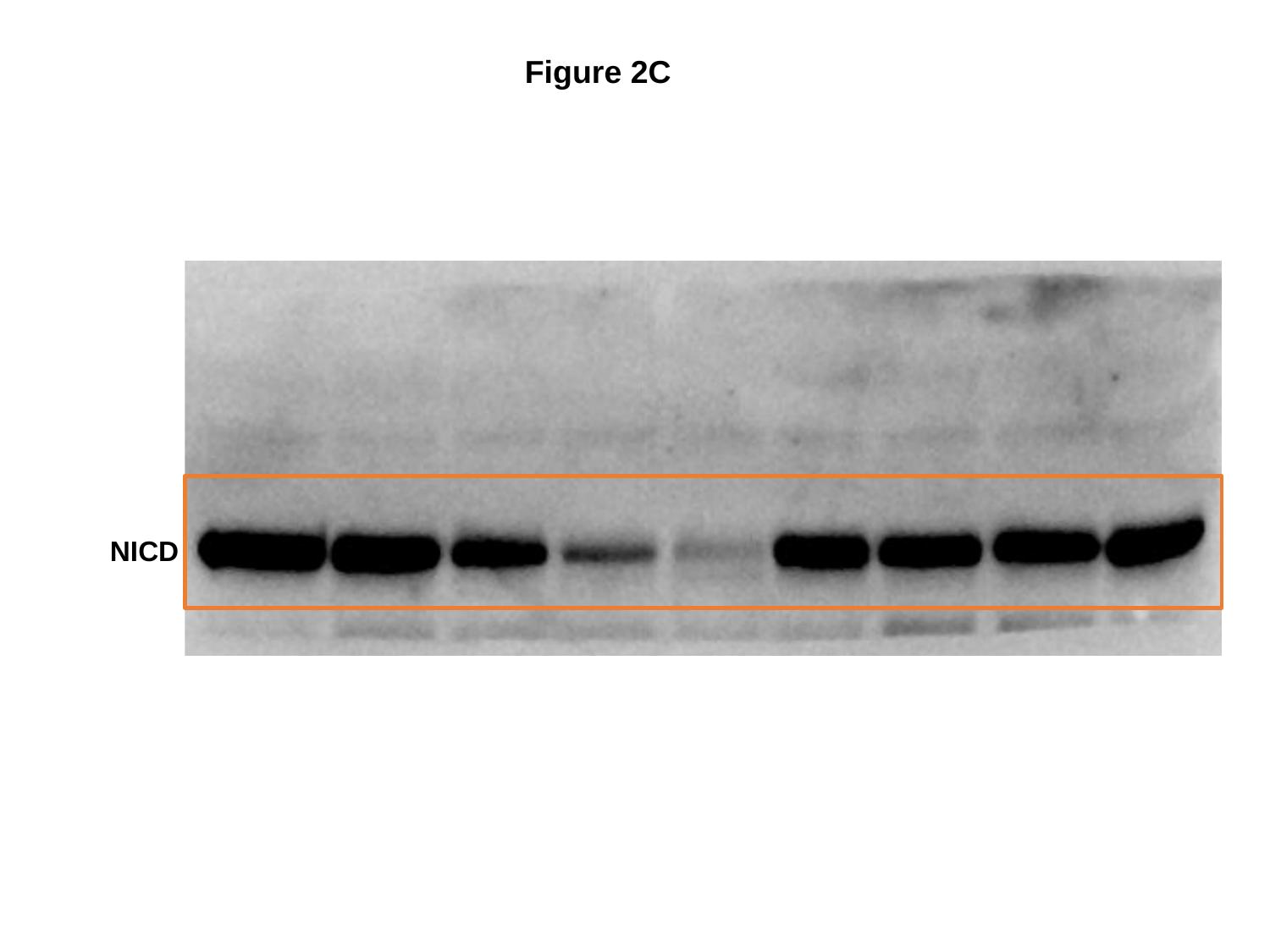

Figure 2C
NICD

## Slide 4
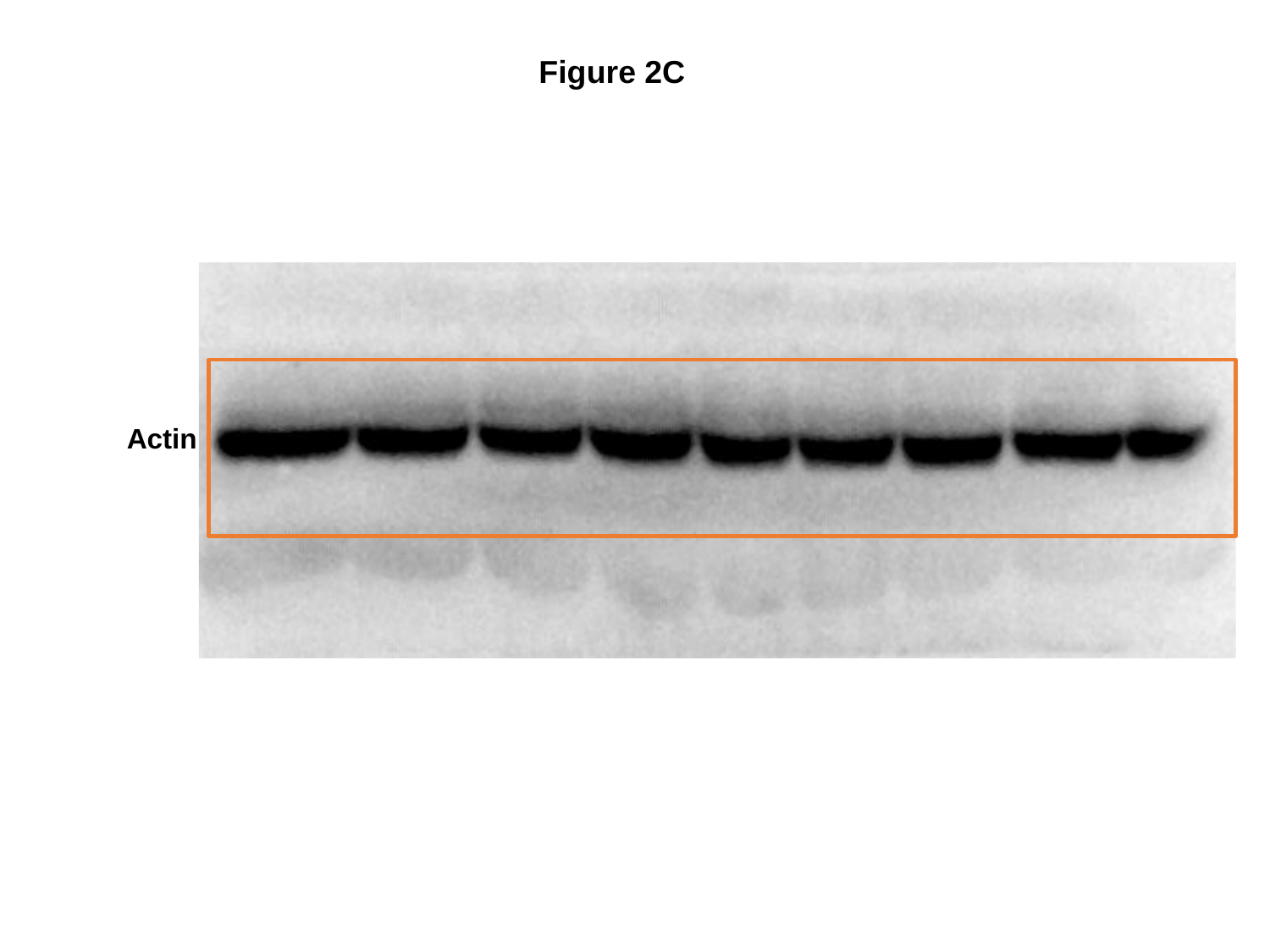

Figure 2C
Actin

## Slide 5
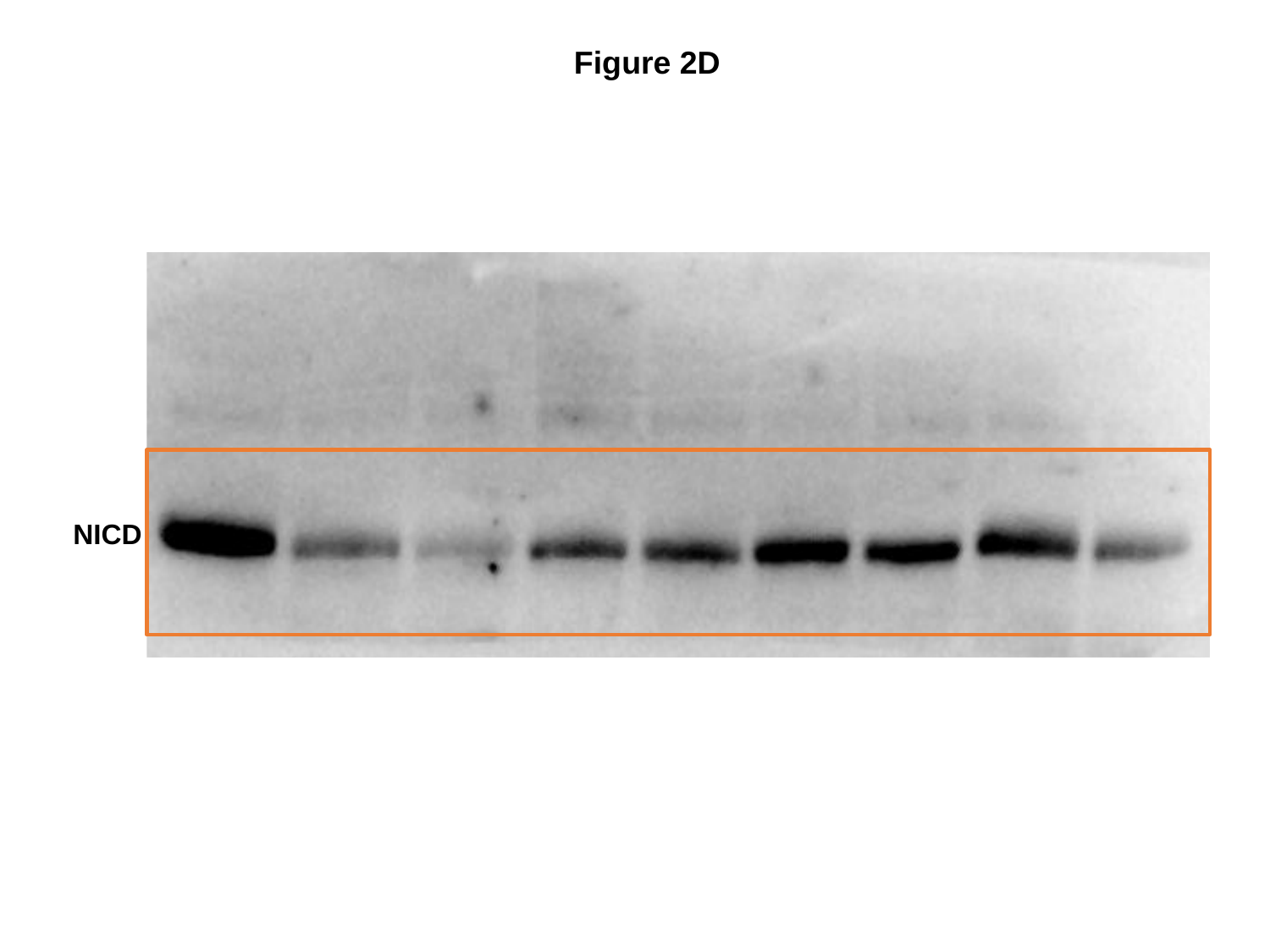

Figure 2D
NICD

## Slide 6
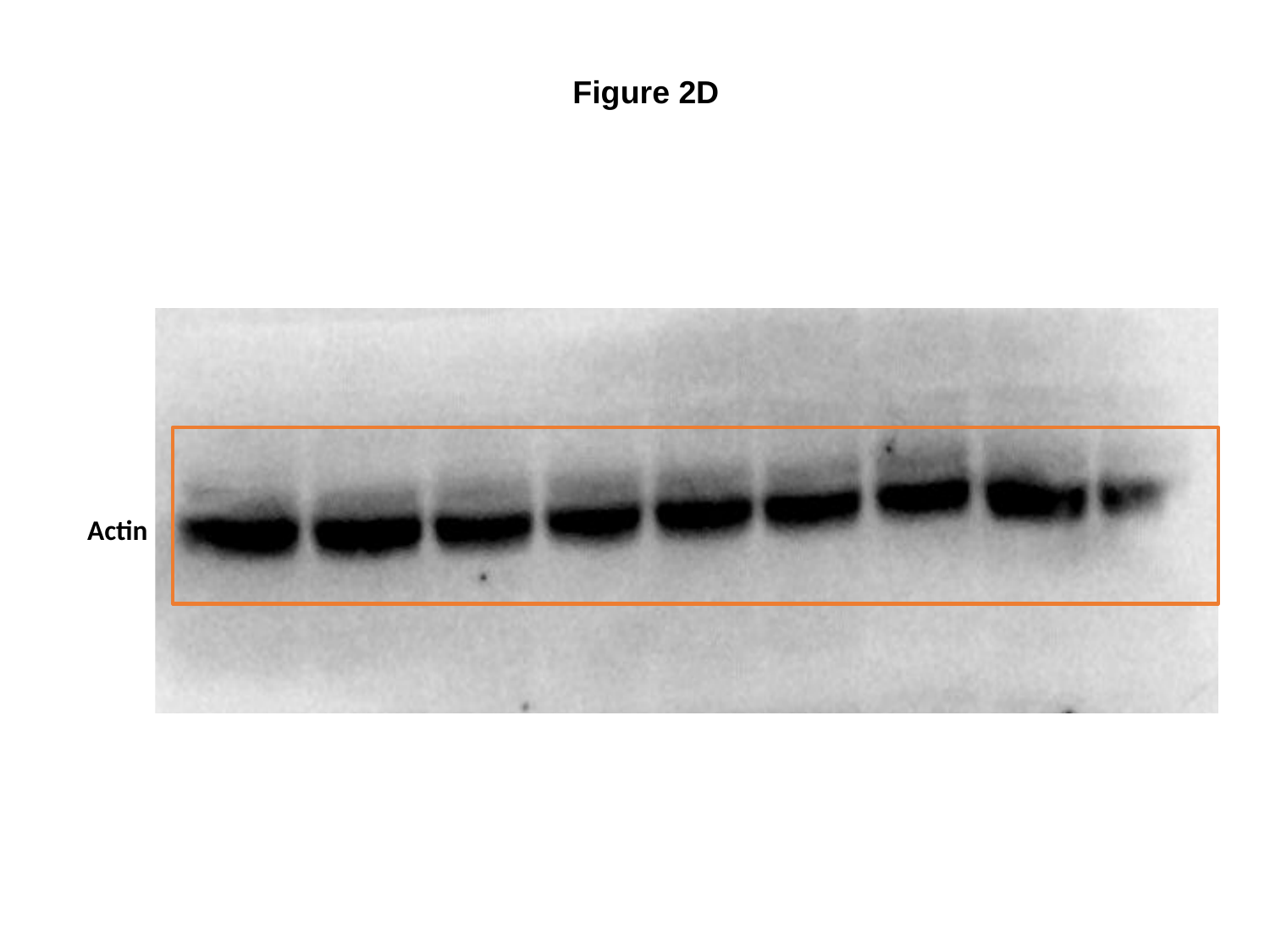

Figure 2D
Actin

## Slide 7
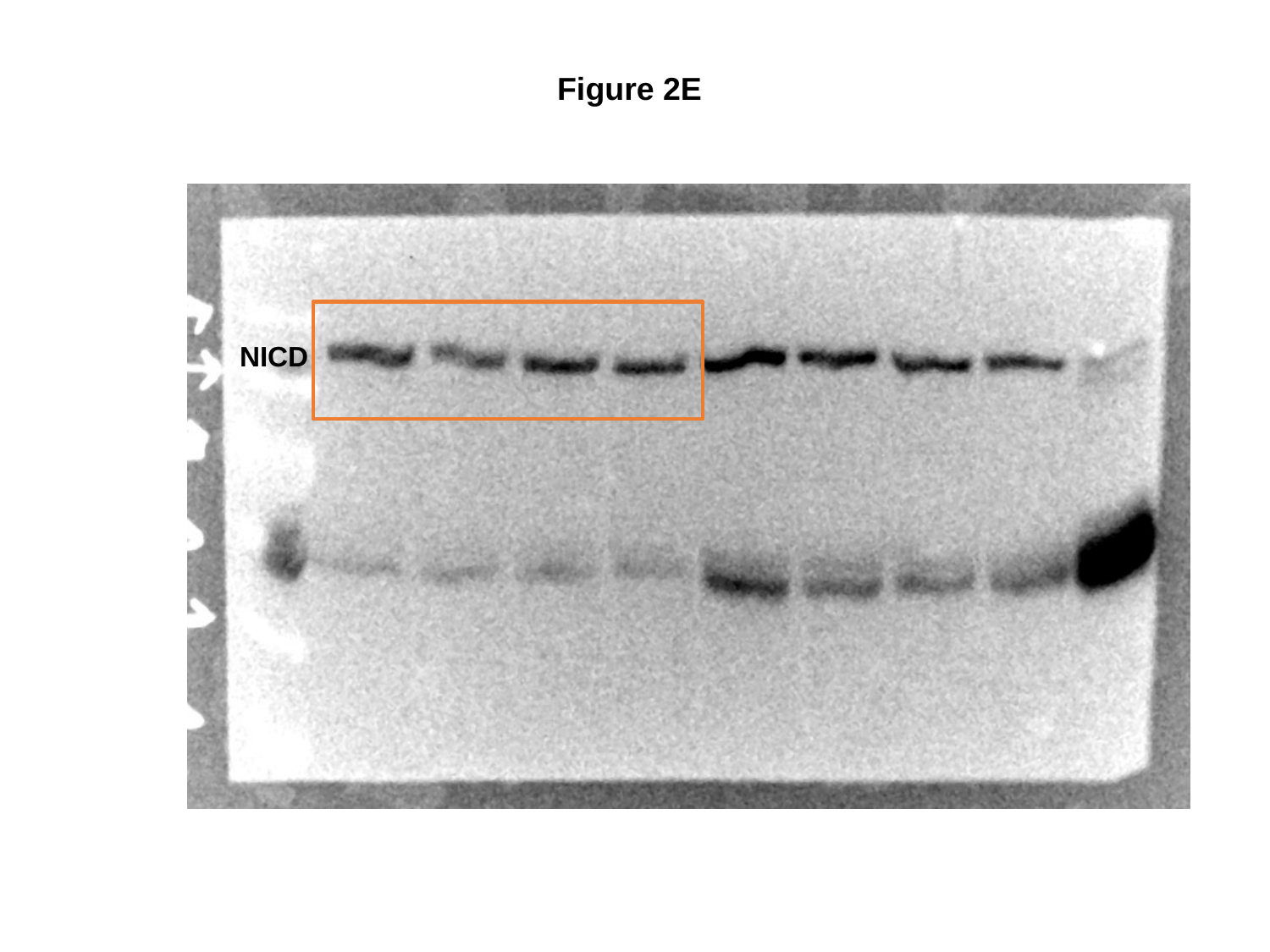

Figure 2E
NICD

## Slide 8
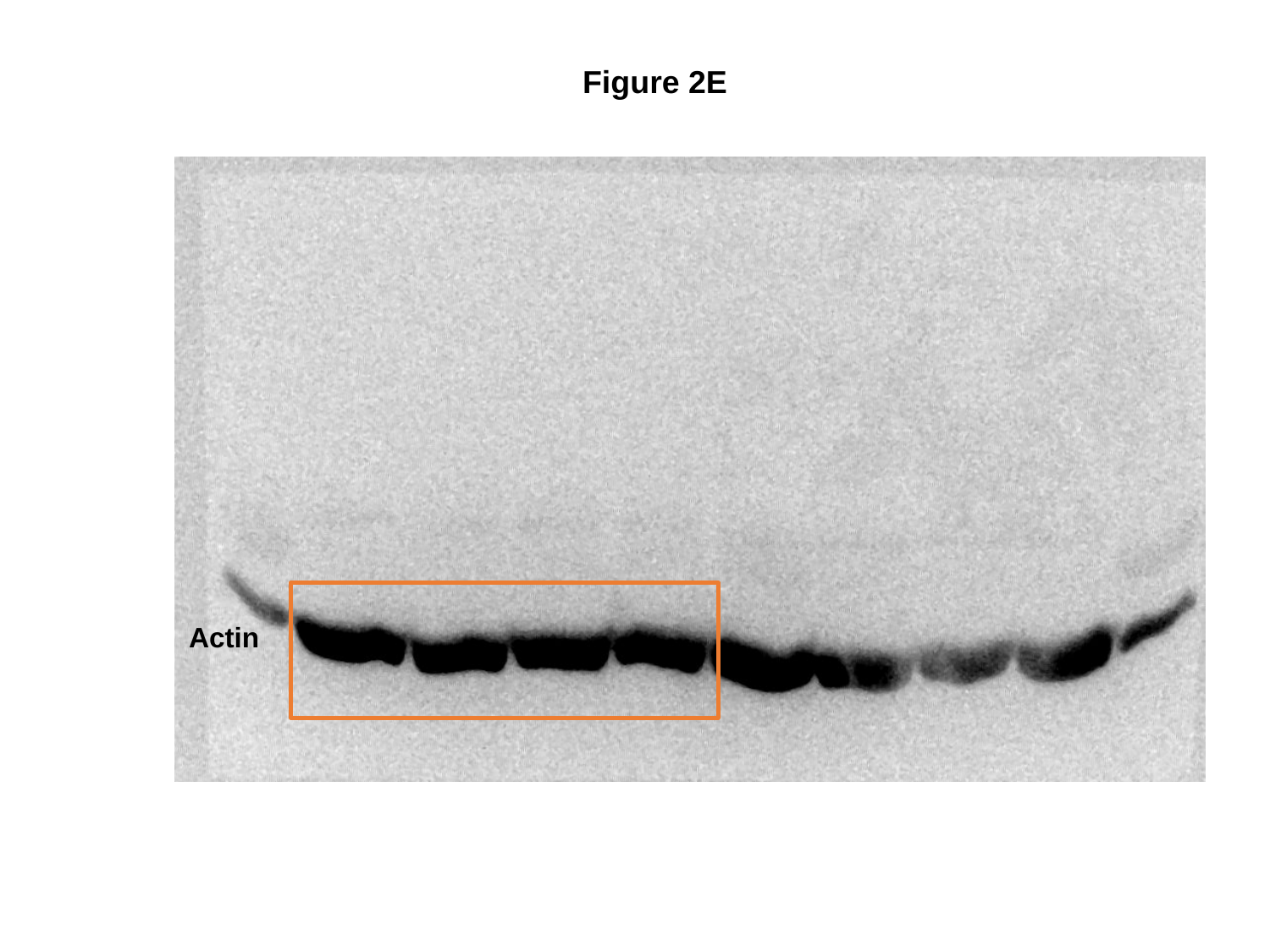

Figure 2E
Actin
