# Supplementary material for: Notch signaling functions in noncanonical juxtacrine manner in platelets to amplify thrombogenicity
Source: eLife. 2022 Oct 3;11:e79590. doi: 10.7554/eLife.79590 (PMC9629830; doi:10.7554/eLife.79590)
Supplement: Figure 3—source data 2. [file elife-79590-fig3-data2.zip › Figure 3 (Labelled blot).pptx]

## Slide 1
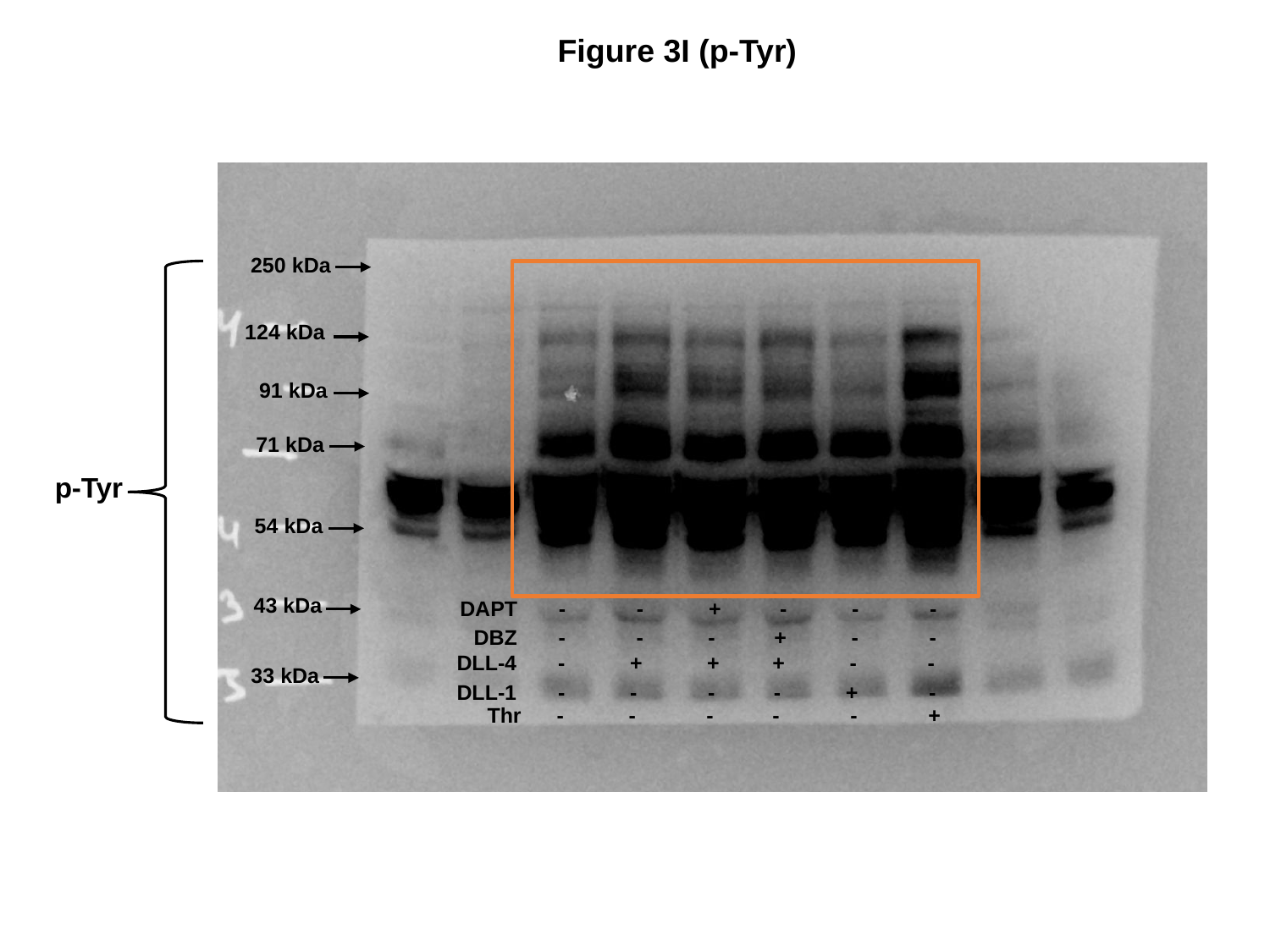

Figure 3I (p-Tyr)
250 kDa
124 kDa
91 kDa
71 kDa
p-Tyr
54 kDa
43 kDa
DAPT - - + - - -
DBZ - - - + - -
DLL-4 - + + + - -
DLL-1 - - - - + -
Thr - - - - - +
33 kDa

## Slide 2
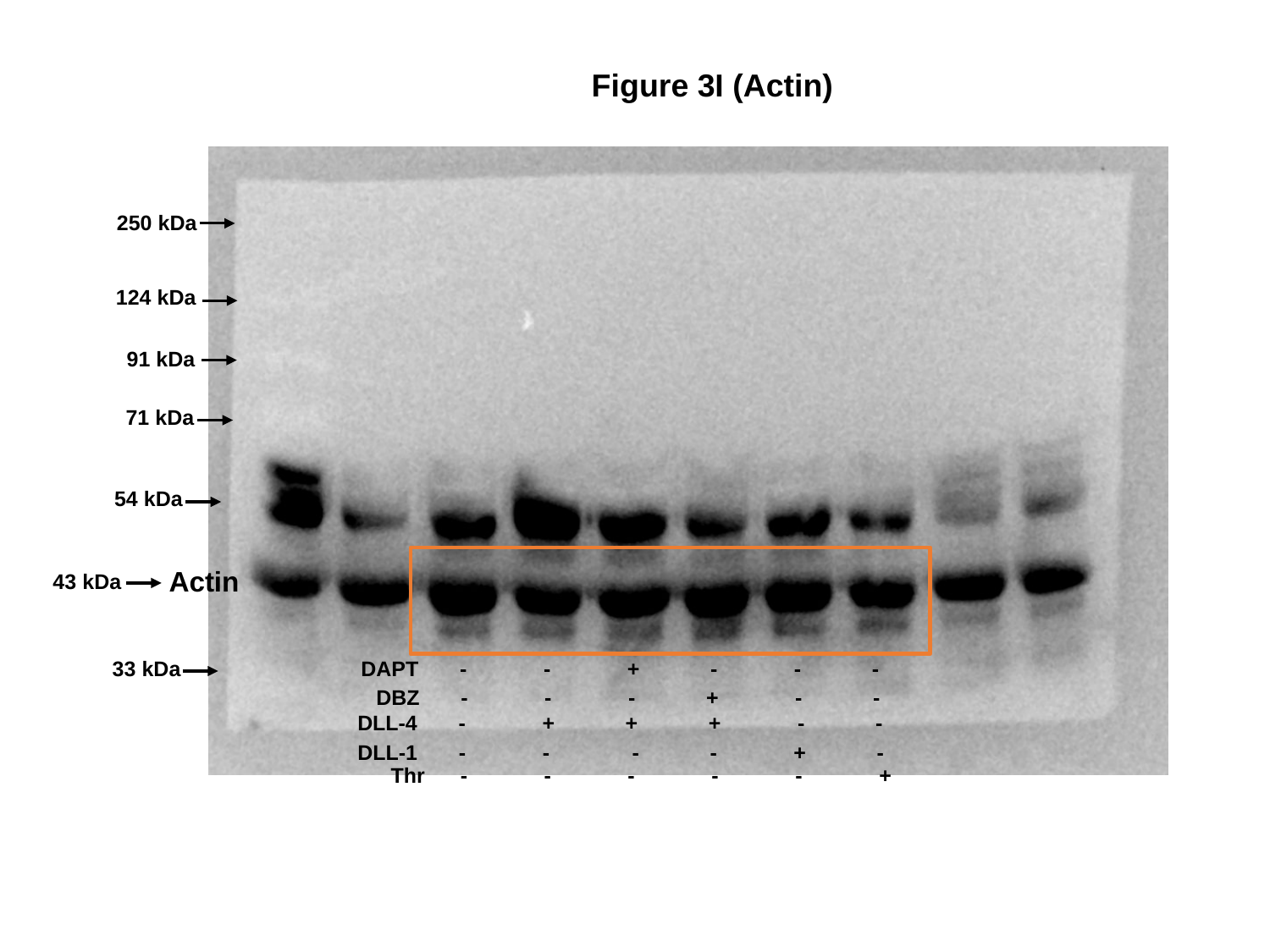

Figure 3I (Actin)
250 kDa
124 kDa
91 kDa
71 kDa
54 kDa
Actin
43 kDa
DAPT - - + - - -
DBZ - - - + - -
DLL-4 - + + + - -
DLL-1 - - - - + -
Thr - - - - - +
33 kDa

## Slide 3
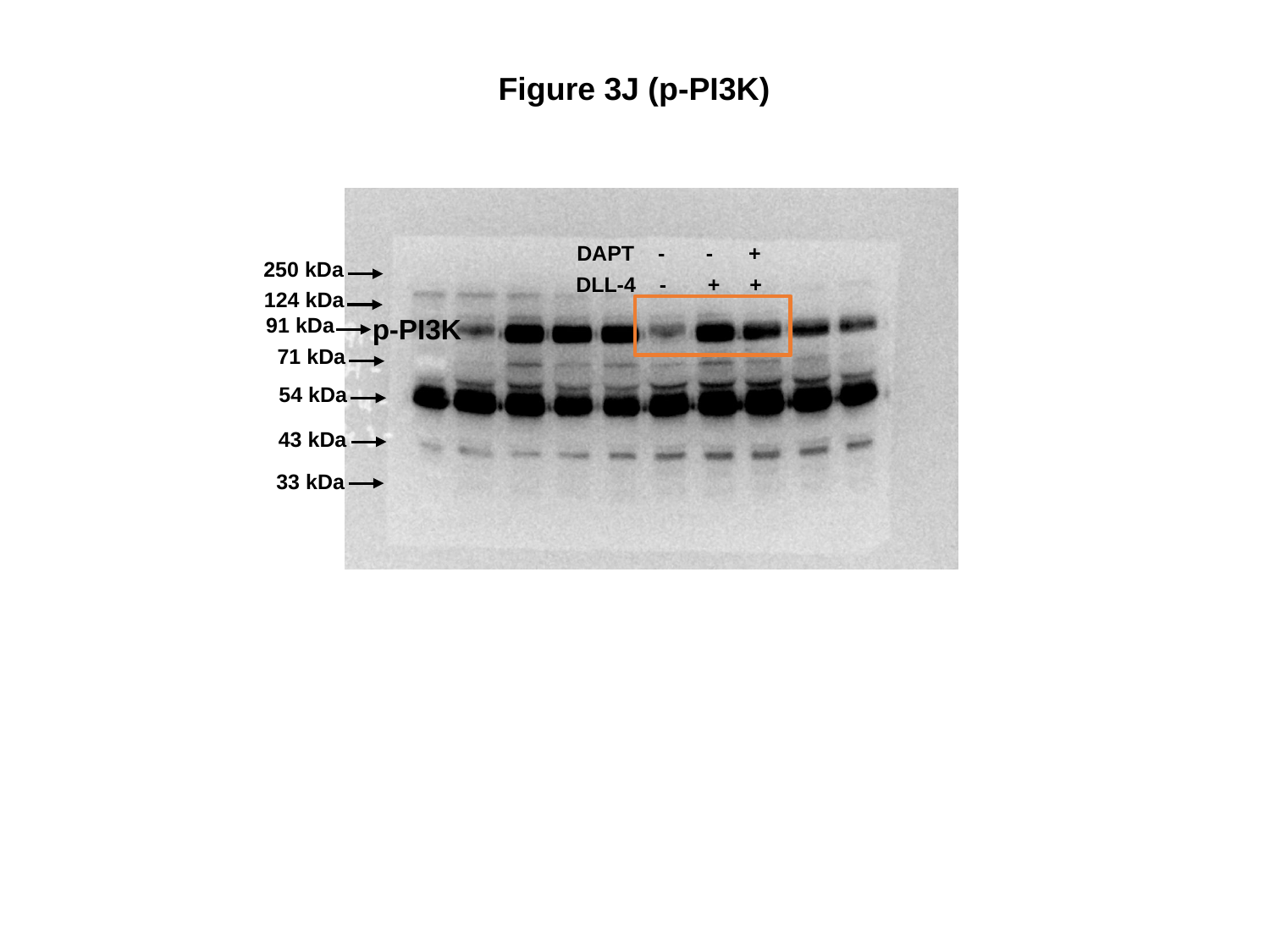

Figure 3J (p-PI3K)
DAPT - - +
DLL-4 - + +
250 kDa
124 kDa
p-PI3K
91 kDa
71 kDa
54 kDa
43 kDa
33 kDa

## Slide 4
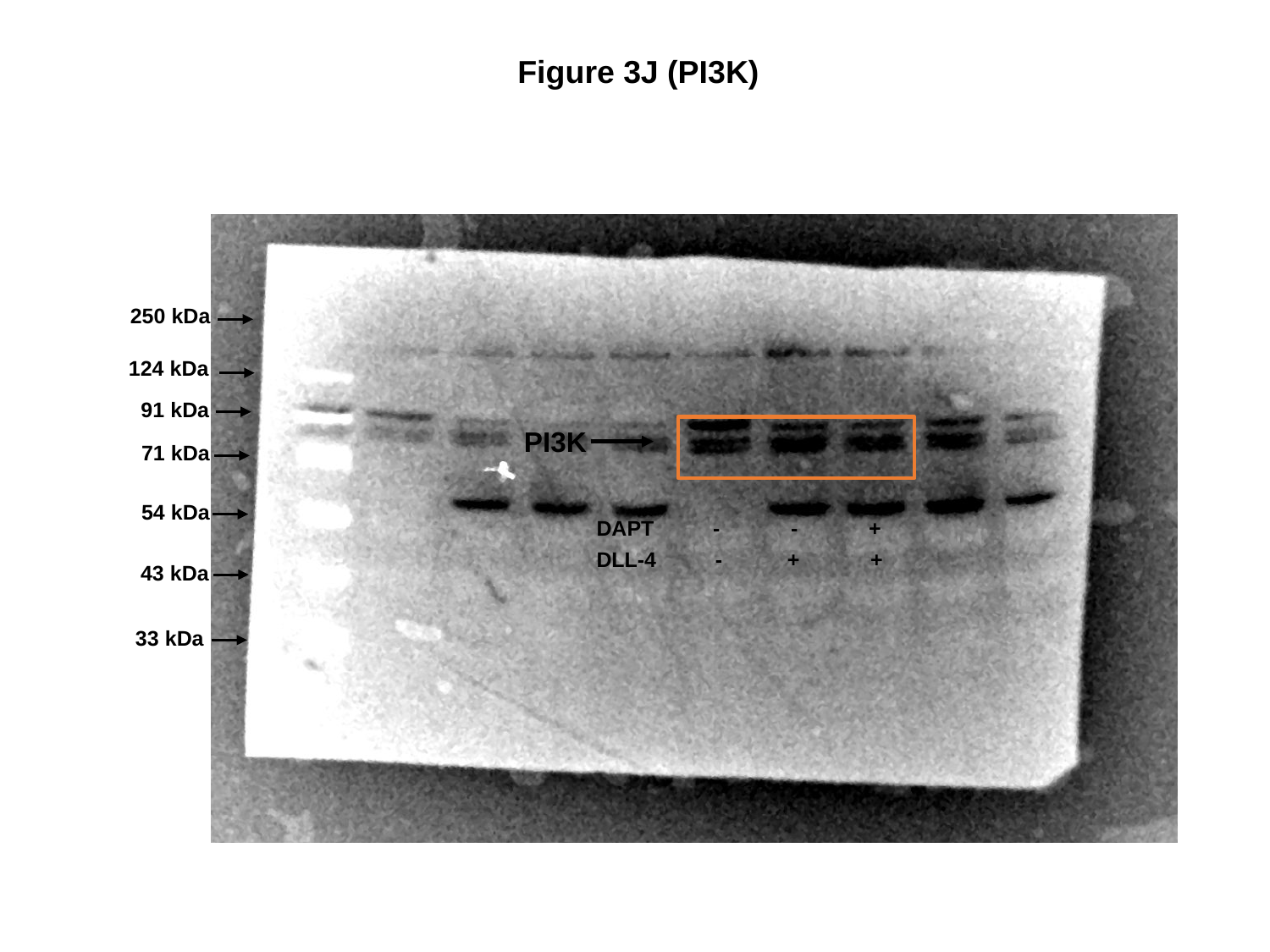

Figure 3J (PI3K)
250 kDa
124 kDa
91 kDa
PI3K
71 kDa
54 kDa
DAPT - - +
DLL-4 - + +
43 kDa
33 kDa

## Slide 5
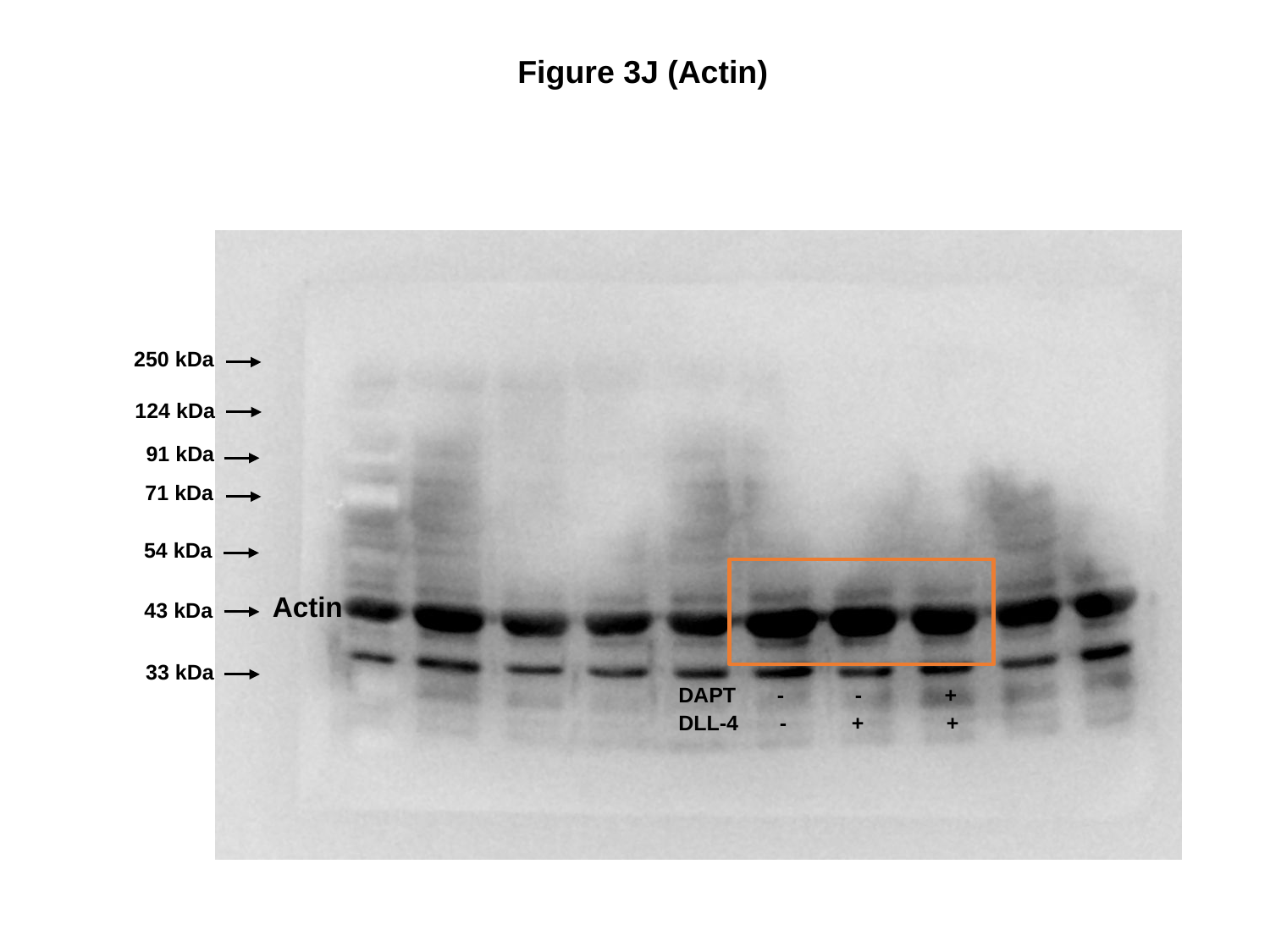

Figure 3J (Actin)
250 kDa
124 kDa
91 kDa
71 kDa
54 kDa
Actin
43 kDa
33 kDa
DAPT - - +
DLL-4 - + +

## Slide 6
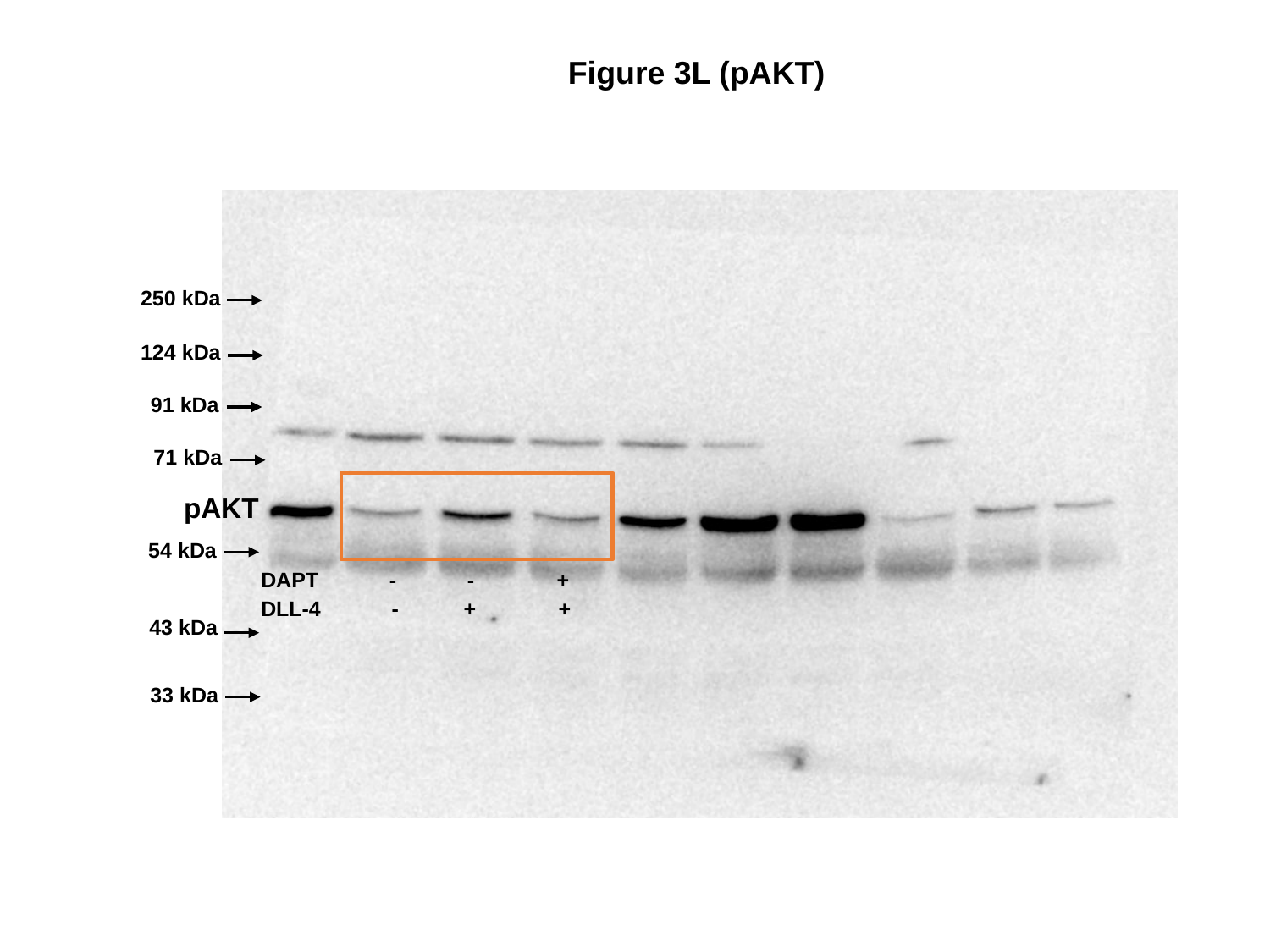

Figure 3L (pAKT)
250 kDa
124 kDa
91 kDa
71 kDa
pAKT
54 kDa
DAPT - - +
DLL-4 - + +
43 kDa
33 kDa

## Slide 7
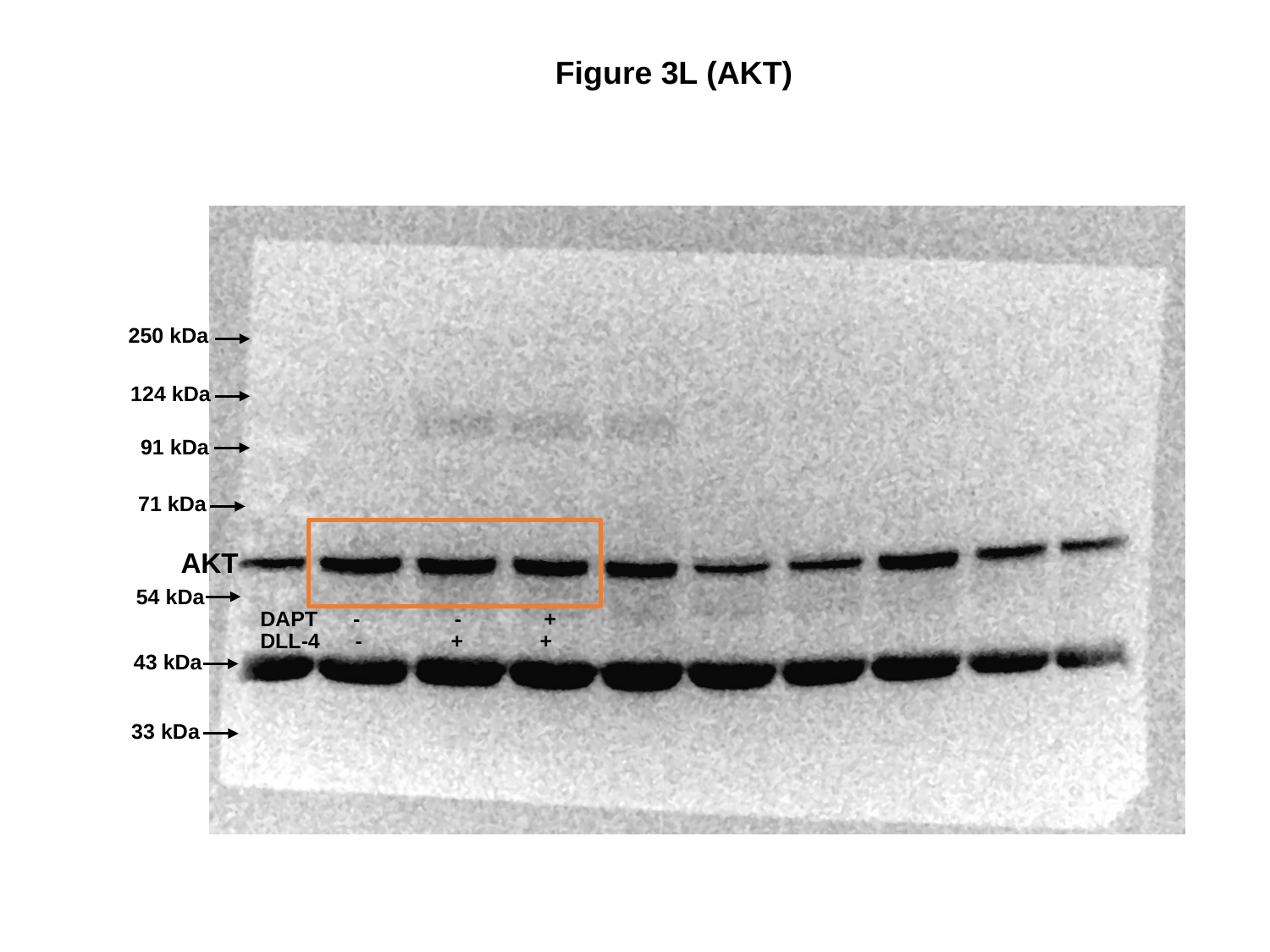

Figure 3L (AKT)
250 kDa
124 kDa
91 kDa
71 kDa
AKT
54 kDa
DAPT - - +
DLL-4 - + +
43 kDa
33 kDa

## Slide 8
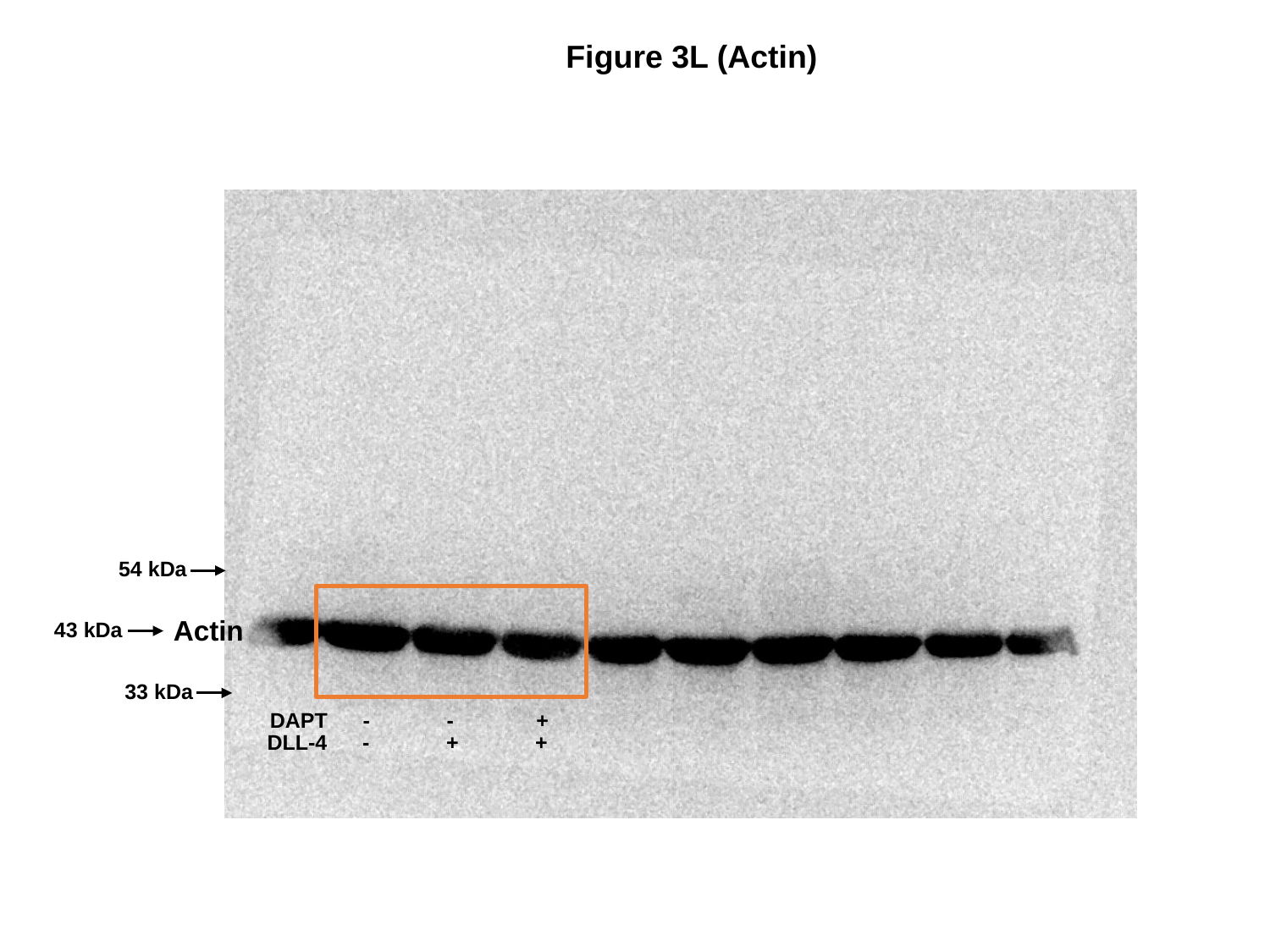

Figure 3L (Actin)
54 kDa
Actin
43 kDa
33 kDa
DAPT - - +
DLL-4 - + +
