# Supplementary material for: Notch signaling functions in noncanonical juxtacrine manner in platelets to amplify thrombogenicity
Source: eLife. 2022 Oct 3;11:e79590. doi: 10.7554/eLife.79590 (PMC9629830; doi:10.7554/eLife.79590)
Supplement: Figure 3—source data 3. [file elife-79590-fig3-data3.zip › Figure 3 (Unedited blot).pptx]

## Slide 1
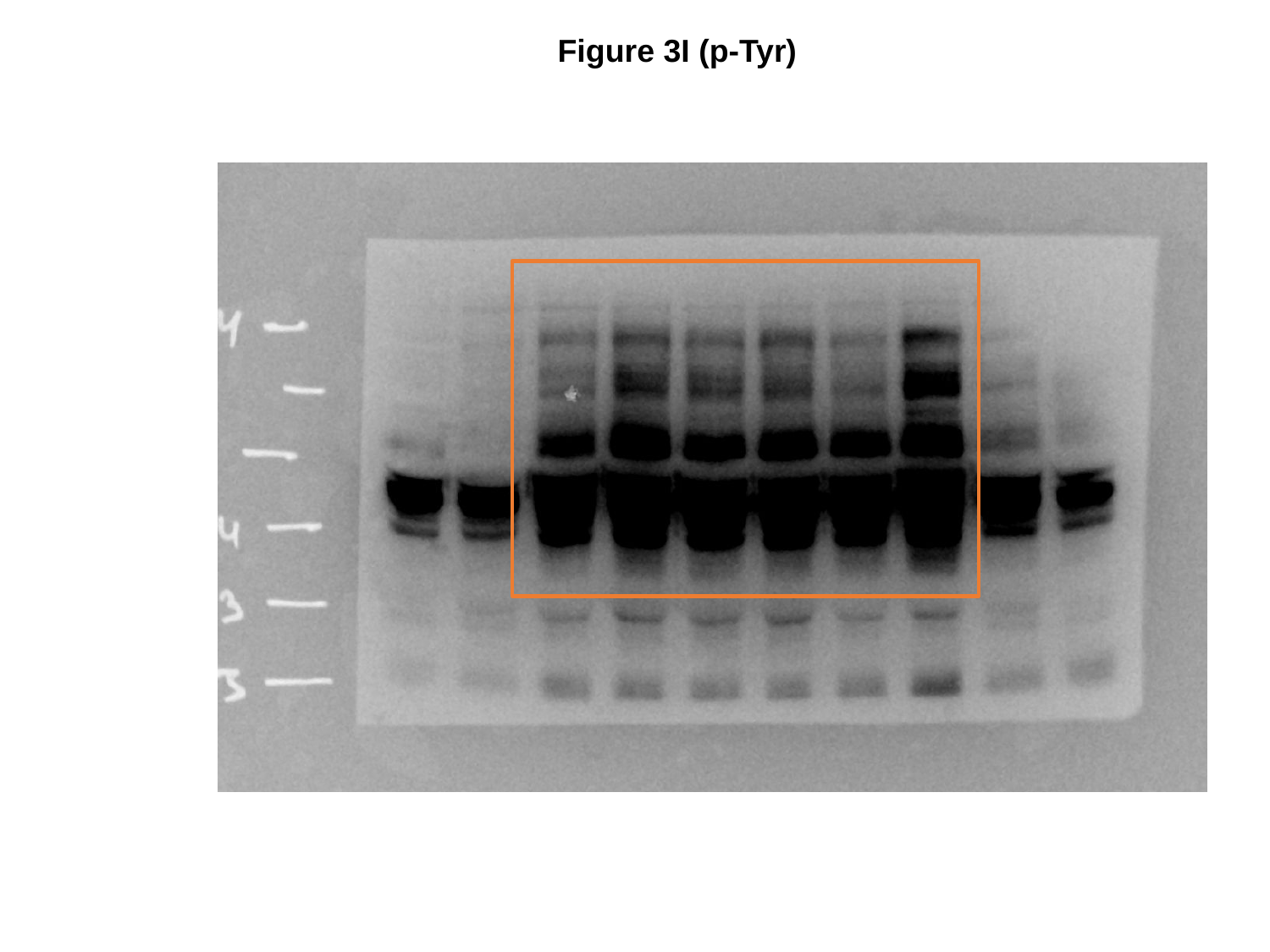

Figure 3I (p-Tyr)

## Slide 2
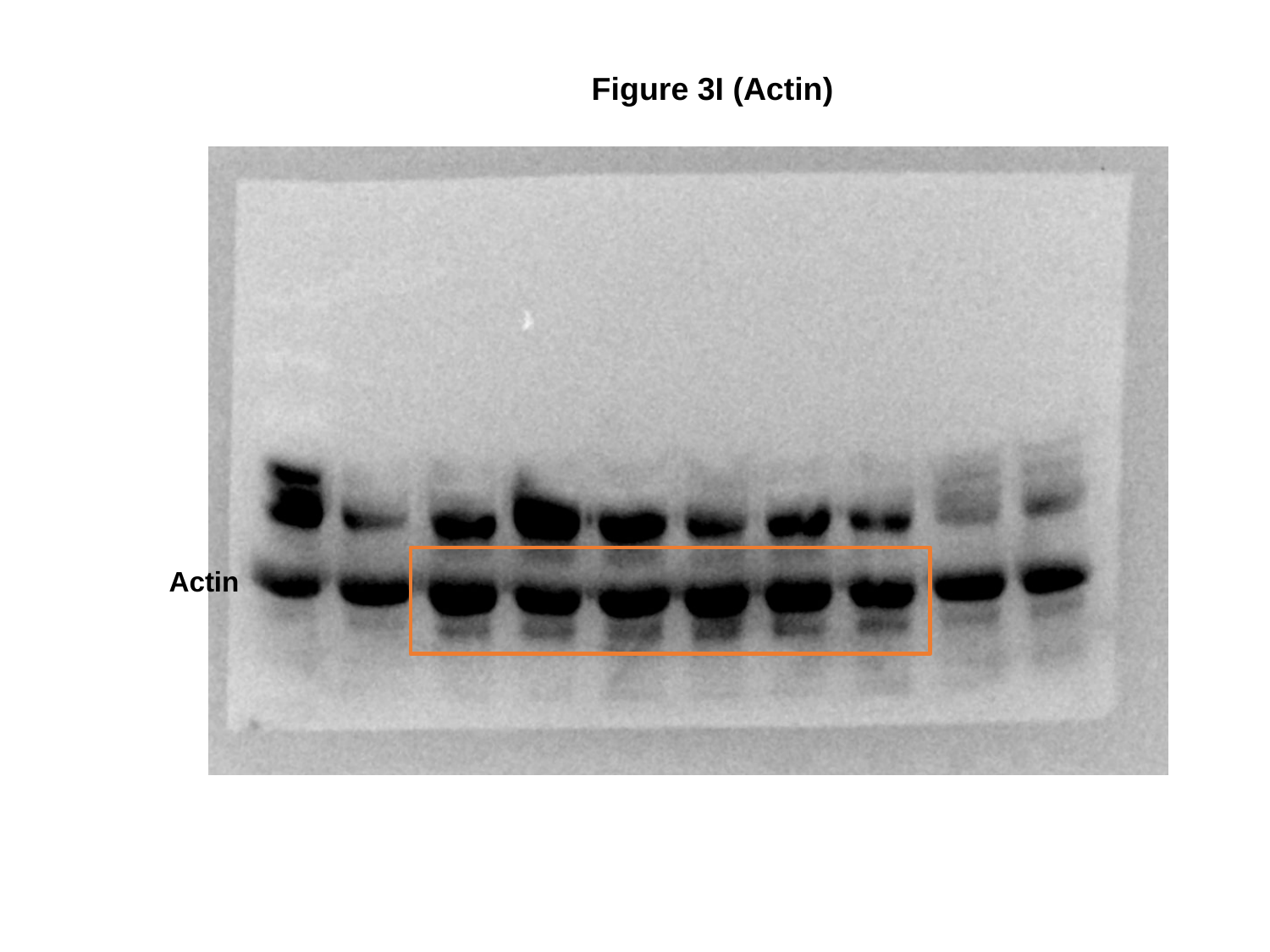

Figure 3I (Actin)
Actin

## Slide 3
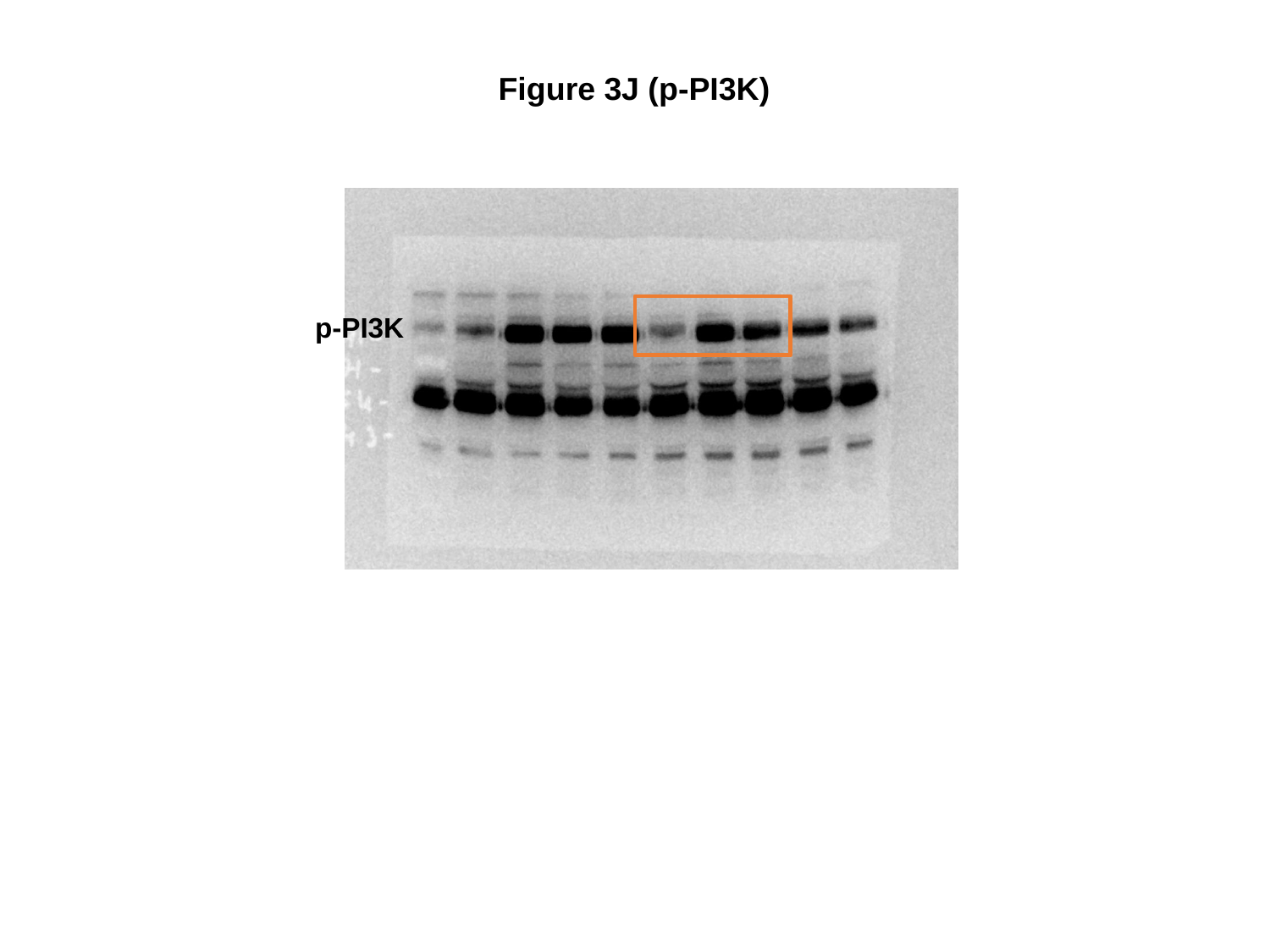

Figure 3J (p-PI3K)
p-PI3K

## Slide 4
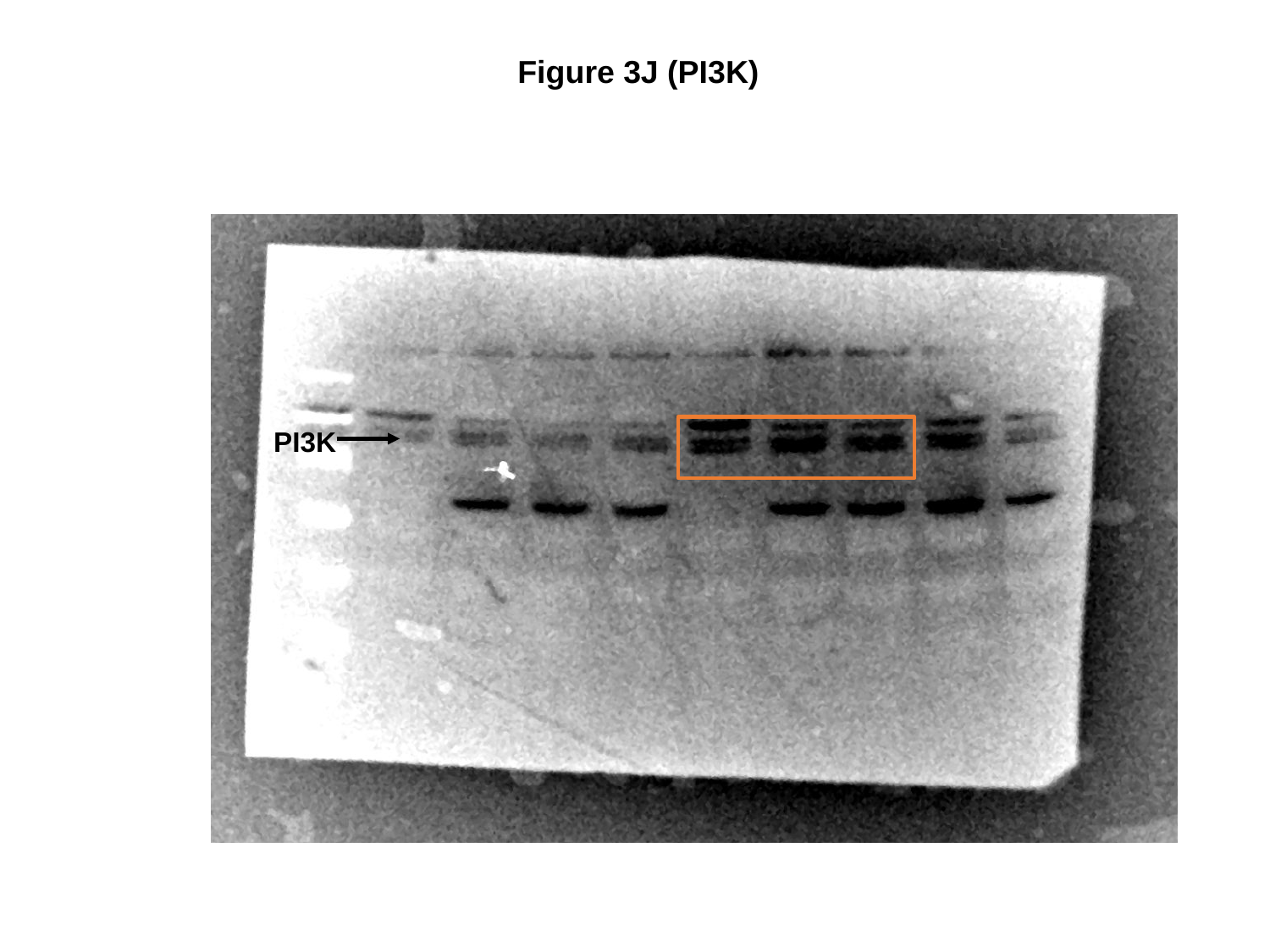

Figure 3J (PI3K)
PI3K

## Slide 5
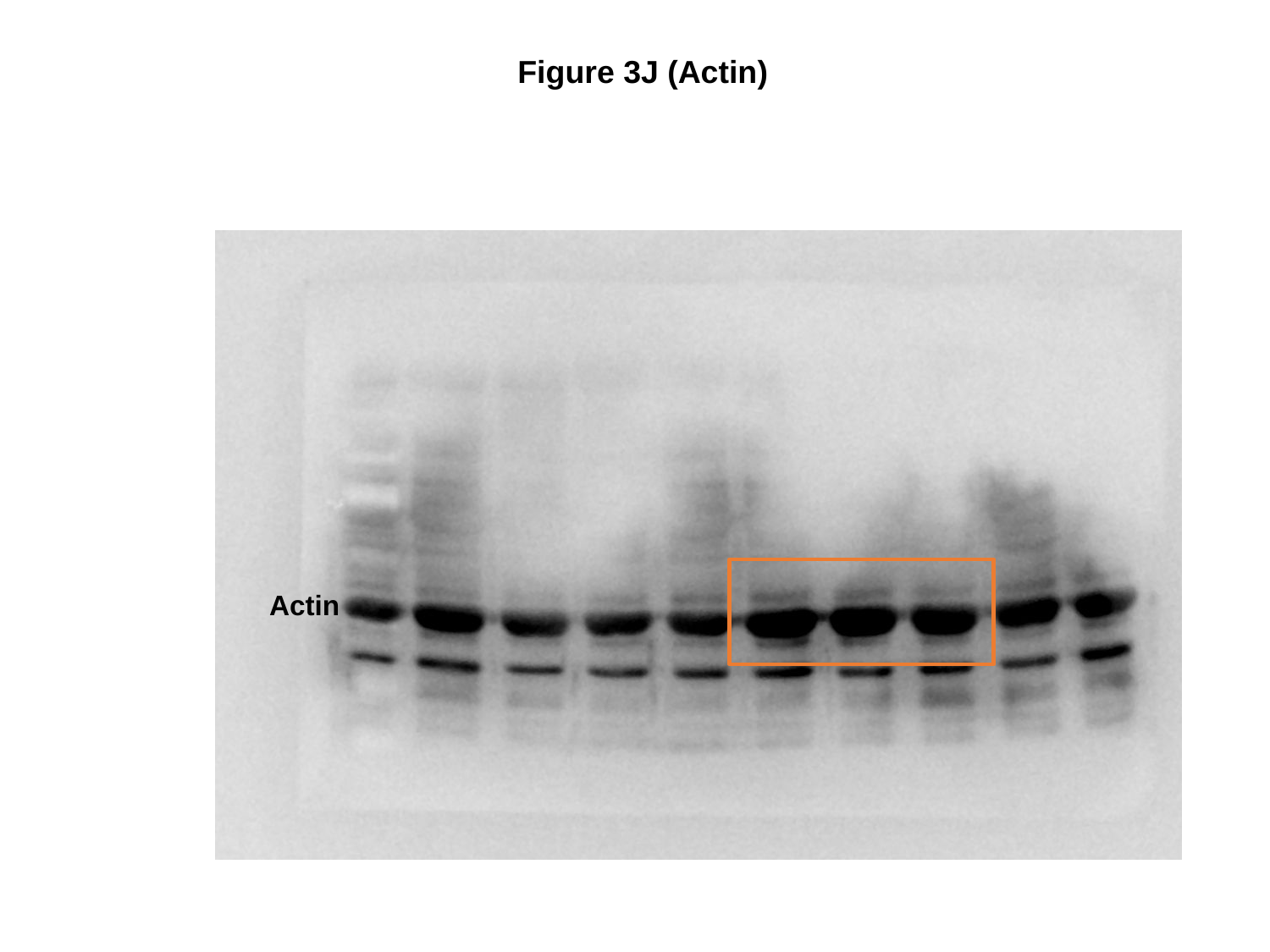

Figure 3J (Actin)
Actin

## Slide 6
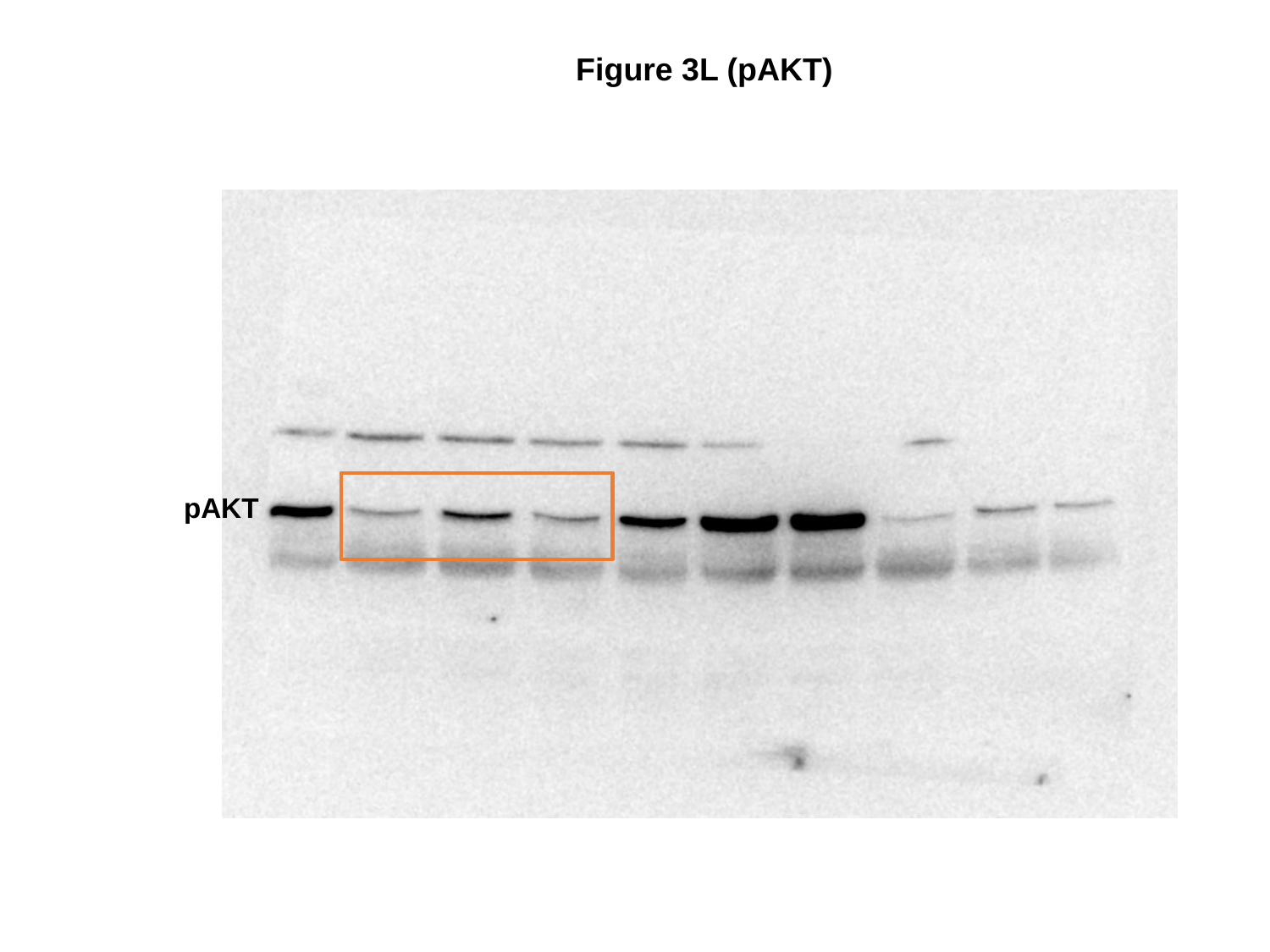

Figure 3L (pAKT)
pAKT

## Slide 7
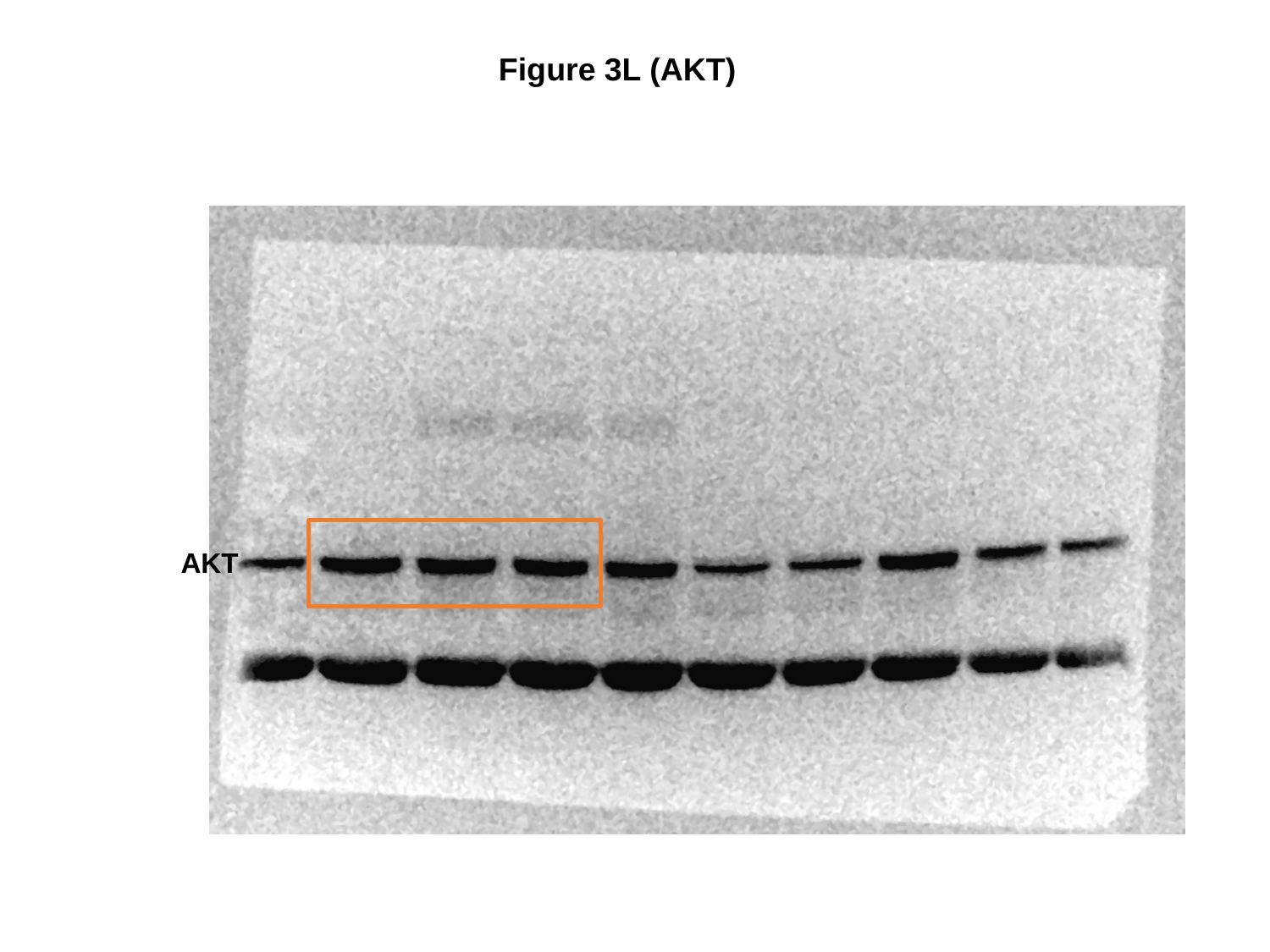

Figure 3L (AKT)
AKT

## Slide 8
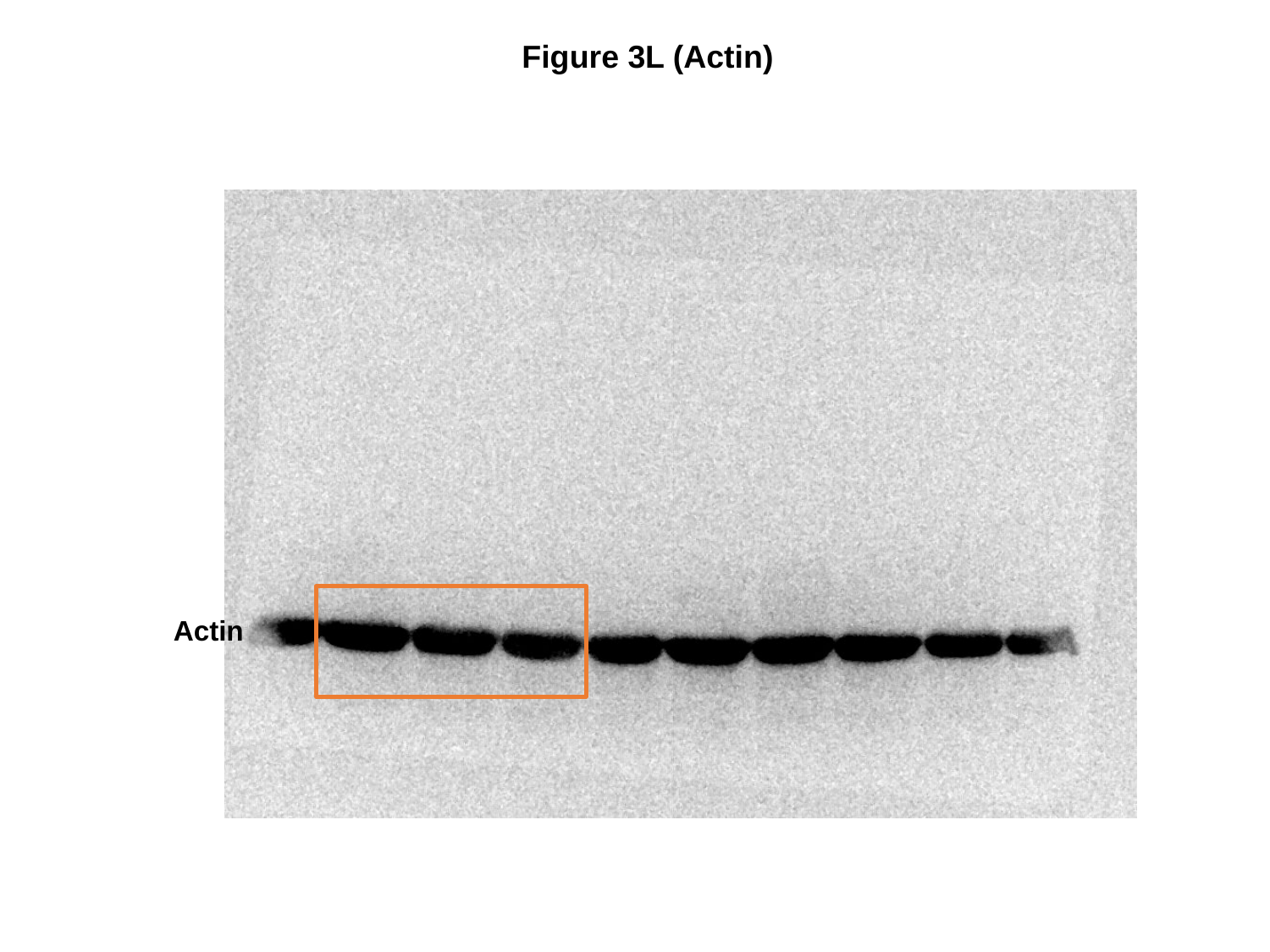

Figure 3L (Actin)
Actin
