# Supplementary material for: Notch signaling functions in noncanonical juxtacrine manner in platelets to amplify thrombogenicity
Source: eLife. 2022 Oct 3;11:e79590. doi: 10.7554/eLife.79590 (PMC9629830; doi:10.7554/eLife.79590)
Supplement: Supplementary file 1. — Data are representative of five individual experiments and presented as mean ± SEM, analyzed by Student’s paired t-test. [file elife-79590-supp1.docx]

|  | **R**  **(Reaction time) (min)** | **K**  **(Clot kinetics)** | **Angle** | **MA**  **(Maximum amplitude) (mm)** |
| --- | --- | --- | --- | --- |
| **Control** | 5.48 (±0.52) | 2.34 (±0.24) | 60.78 (±2.56) | 62.44 (±2.25) |
| **DAPT** | 7.28 (±1.06) | 2.56 (±0.26) | 55.96 (±2.38) | 57.8 (±2.54) |
| **P value** | 0.0432 | 0.5388 | 0.1707 | 0.0046 |
